# Study of Endogen Substrates, Drug Substrates and Inhibitors Binding Conformations on MRP4 and Its Variants by Molecular Docking and Molecular Dynamics

**DOI:** 10.3390/molecules26041051

**Published:** 2021-02-17

**Authors:** Edgardo Becerra, Giovanny Aguilera-Durán, Laura Berumen, Antonio Romo-Mancillas, Guadalupe García-Alcocer

**Affiliations:** 1 Posgrado en Ciencias Químico Biológicas, Facultad de Química, Universidad Autónoma de Querétaro, Cerro de las Campanas S/N, Querétaro 76010, Mexico; ebecerra1989@gmail.com (E.B.); giovanny.aguilera@uaq.mx (G.A.-D.); 2 Centro Universitario, Unidad de Investigación Genética, Facultad de Química, Universidad Autónoma de Querétaro, Querétaro 76010, Mexico; lcbsq@yahoo.com; 3 Centro Universitario, Laboratorio de Diseño Asistido por Computadora y Síntesis de Fármacos, Facultad de Química, Universidad Autónoma de Querétaro, Querétaro 76010, Mexico

**Keywords:** MRP4, SNPs, variants, protein threading modeling, molecular docking, molecular dynamics, binding site

## Abstract

Multidrug resistance protein-4 (MRP4) belongs to the ABC transporter superfamily and promotes the transport of xenobiotics including drugs. A non-synonymous single nucleotide polymorphisms (nsSNPs) in the ABCC4 gene can promote changes in the structure and function of MRP4. In this work, the interaction of certain endogen substrates, drug substrates, and inhibitors with wild type-MRP4 (WT-MRP4) and its variants G187W and Y556C were studied to determine differences in the intermolecular interactions and affinity related to SNPs using protein threading modeling, molecular docking, all-atom, coarse grained, and umbrella sampling molecular dynamics simulations (AA-MDS and CG-MDS, respectively). The results showed that the three MRP4 structures had significantly different conformations at given sites, leading to differences in the docking scores (DS) and binding sites of three different groups of molecules. Folic acid (FA) had the highest variation in DS on G187W concerning WT-MRP4. WT-MRP4, G187W, Y556C, and FA had different conformations through 25 ns AA-MD. Umbrella sampling simulations indicated that the Y556C-FA complex was the most stable one with or without ATP. In Y556C, the cyclic adenosine monophosphate (cAMP) and ceefourin-1 binding sites are located out of the entrance of the inner cavity, which suggests that both cAMP and ceefourin-1 may not be transported. The binding site for cAMP and ceefourin-1 is quite similar and the affinity (binding energy) of ceefourin-1 to WT-MRP4, G187W, and Y556C is greater than the affinity of cAMP, which may suggest that ceefourin-1 works as a competitive inhibitor. In conclusion, the nsSNPs G187W and Y556C lead to changes in protein conformation, which modifies the ligand binding site, DS, and binding energy.

## 1. Introduction

The transport of xenobiotics out of the cell across membranes is a mechanism used by cells to detoxify. This mechanism is mediated by ATP-binding cassette (ABC) transporters [1]. Multidrug resistance protein-4 (MRP4) is a member of the ABCC subfamily and mediates the transport of xenobiotics such as cardiovascular, antiviral, and anticancer drugs. The substrates for MRP4 are mainly glucuronide conjugates and organic anions [2]. MRP4 can modify drug pharmacokinetics and contributes to the manifestation of side effects or multidrug resistance. In addition, the tumor energy metabolism is related to multidrug resistance due to the high production of ATP to enhance the activity of MRP4 and other ABC transporters [3]. An increase in MRP4 activity or expression leads to a decrease in drug efficacy. In another instance, a decrease in MRP4 activity or expression could enhance toxicity due to drug accumulation. Since MRP4 is expressed in the kidneys, liver, erythrocytes, lymphocytes, adrenal glands, platelets, brain, and pancreas in humans, it can modify cellular exposure to drugs. In addition, the toxicity produced by MRP4 will depend on the type of drug or endogen substrate [4]. The MRP4 dysregulation has been reported in several pathological disorders, especially in cancer [5]; thus, MRP4 represents an attractive therapeutic target. The design of pharmacological agents with the ability to selectively modulate the activity of this ABC transporter or modify its affinity of a given substrate represents a challenge in chemical biology and drug design [6,7].

MRP4 consists of 1325 amino acids and is the shortest member of the ABCC subfamily [8]. The basic MRP4 core structure is comprised of transmembrane domains (TMDs) and intracellular nucleotide binding domains (NBDs). The domain arrangement for MRP4 is TMD-NBD-TMD-NBD [9]. Each TMD consists of six transmembrane helixes (TMHs) that determine the ligand specificity and allow ligand binding. In addition, NDBs bind and hydrolyze ATP to trigger substrate transport [10]. MRP4 is codified by the ABCC4 gen, located on chromosome 13q32.1 [11]. Alternative splicing leads to four isoforms, of which isoform 1 has been the most studied [1]. MRP4 is a highly polymorphic gene [12]; however, limited data are available on the function of MRP4 variants. Recent studies have been focused on the relationship between ABCC4 nsSNPs and drug disposition. In most cases, nsNSPs have little or no effect on the protein structure or function, but sometimes nsSNPs promotes non-functional or highly functional proteins [13]. The nsSNPs that occur in protein coding regions always alter the encoded amino acid, and the effect on the structure or function of the protein depends on the mutated site [14].

**Figure 1:**
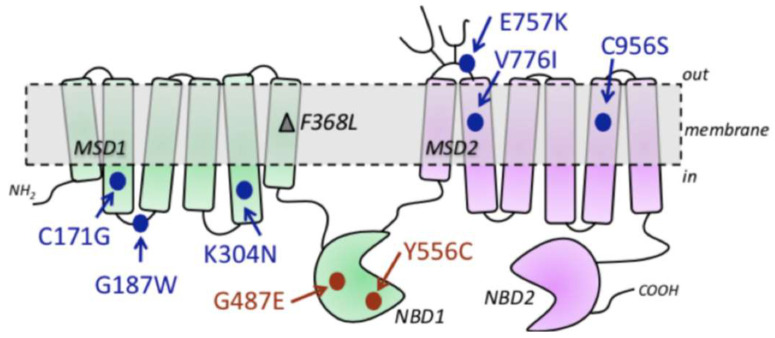
MRP4 structure of the cell membrane and localization of MRP4 variants (adapted from Banerjee at al., 2016) [16].

Nada-Abla and coworkers in 2008 reported that the MRP4 variants G187W and G487E show a significantly reduced function of azidothymidine and adefovir transport compared to wild-type MRP4 (WT-MRP4). G187W is a non-synonymous ABCC4 variant and the mutation is located at the cytosolic loop 1 in the TMD (Figure 1); it has undergone the greatest structural change in terms of composition, polarity, and molecular volume. G187W also has a 50% reduction in function, and this could be clinically relevant [15]. On the other hand, Y556C is another non-synonymous ABCC4 variant and is located at NBD1 (Figure 1). Mayukh-Banerjee and coworkers in 2016 reported that the Y556C variant exhibited a 1.8-fold increase in dimethylarsinic acid effectiveness relative to WT-MRP4. Experiments on MRP4 transfection into the HEK cell line showed that the Y556C variant had 50% less expression than WT-MRP4. Both G487E and Y556C had appropriate cellular membrane localization [16].

The crystallographic structure of MRP4 is not available; thus, a protein threading model can be built based on the homology of the template and the construction of loops. Protein threading by I-TASSER relies in the identification of structural templates from the Protein Data Bank (PDB) using a local meta-threading server, a method for template-based protein structure prediction. Three-dimensional models are generated for a given sequence by collecting high-scoring structural templates from locally installed threading programs [17,18]. After model building, it can be refined by molecular mechanics calculations, such as energy minimization and molecular dynamics simulations. Protein threading models are considered working tools that can be used to generate hypotheses related to protein structure, protein function, and protein–ligand interactions. The molecular docking of drug molecules into their binding sites allows us to identify relevant amino acids for ligand–protein interactions in order to select such amino acids for further site-directed mutagenesis studies [9]. In the present study, three different MRP4 structures (WT and its variants, G187W and Y556C) were built through protein threading to study, by molecular docking, the interactions between endogen substrates, drug substrates, and inhibitors, with MRP4-WT, G187W, and Y556C, allowed to observe changes in the pattern of intermolecular interactions, adapted from Russel and coworkers in 2008, where they report the IC_50_ of several molecules, in vitro, over the MRP4 protein [8], and to calculate the binding energy (ΔG) between FA and cAMP and MRP4 structures.

## 2. Results and Discussion

### 2.1. WT-MRP4, G187W, and Y556C Model Building and CG-MD Simulations

MRP4 plays a critical role in the distribution of different xenobiotics and endogen substrates, which can lead to different effects in the organism. Differences in the MRP4 activity depend, among other factors, on the expression or mutational changes of the ABCC4 gene, leading to significantly higher or lower transport activity [4]. The WT-MRP4 and its variants Y556C and G187W were built by protein threading in the I-TASSER server [19]. Protein threading and homology modeling are based on the principle that similar primary sequences will lead to similar 3D protein structures. According to the BLAST server, the template structure MRP1 from *Bos taurus* and human MRP4 had a 36.56% identity sequence similarity. When the primary sequence of a protein has 30% of identity as referred to a template (crystallographic structure), the protein threading and homology models are considered functional because the root mean standard deviation (RMSD) of the positions of their atoms is 2.0 Å or less with regard to the template structure [20,21,22].

**Figure 2:**
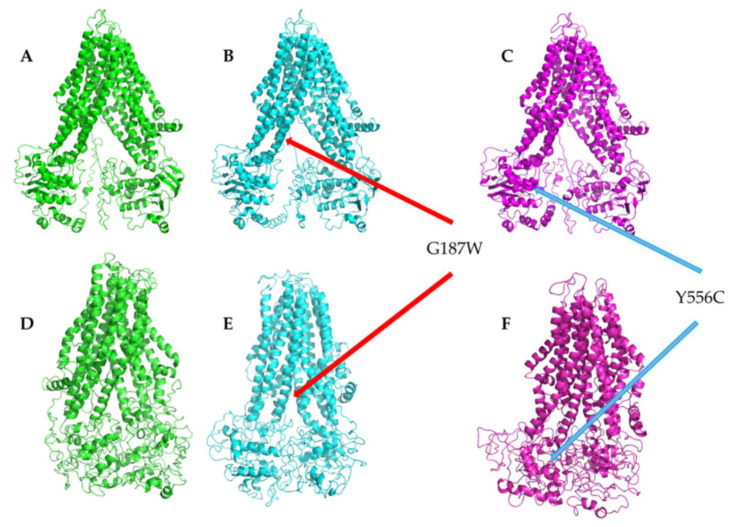
MRP4 models built by homology modeling in I-TASSER and cluster 1 from CG-MDS. Green, (**A**) WT-MRP4. Cyan, (**B**) G187W. Magenta, (**C**) Y556C. (**D**–**F**) represent cluster 1 obtained from GC-MDS for WT-MRP4, G187W, and Y556C, respectively. The arrows indicate the location of mutations.

The best model by the I-TASSER of each MRP4 structure was selected for further analysis with coarse-grained molecular dynamics simulations (CG-MDS) of 1 μs. Figure 2 shows the three MRP4 models and the most representative structures (cluster 1) obtained in I-TASSER and by CG-MDS at timesteps 630.40 ns for WT-MRP4, 564.90 ns for G187W, and 674.90 ns for Y556C. The conformations of WT-MRP4 and variants were in an “inward-facing conformation” [23], while, in CG-MDS, the three MRP4 structures were in a closed state. All the loops that connect the alpha helixes of the three MRP4 structures have different conformations and distributions over the protein.

**Figure 3:**
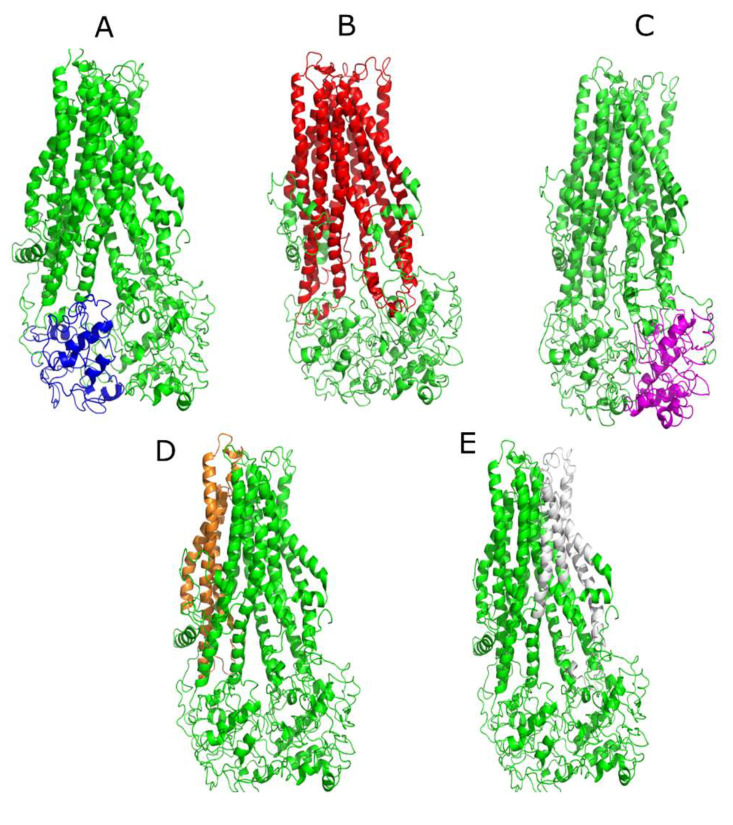
Representation of different sites of the MRP4 protein in the complete structure. (**A**) Blue represents NBD1. (**B**) Red represents TMDs. (**C**) Magenta represents NBD2. (**D**) Orange represents r85-236. (**E**) Gray represents r715-866.

In this work, RMSD values higher than 2.0 Å were considered significant, hence the protein conformations were considered different. Figure 3 shows the different MRP4 sites studied and each region is illustrated with a different color, where the green color represents WT-MRP4; those sites are the nucleotide-binding domains (NBD), the transmembrane domains (TMD), and the residues relevant to substrate interaction (r85-236 and r715-866).

**Figure 4:**
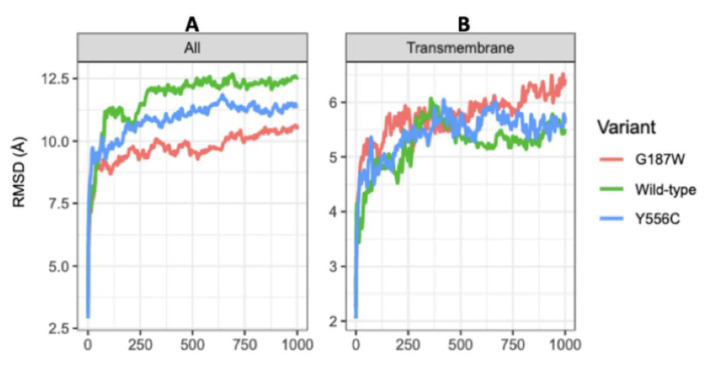
plot for the complete WT-MRP4 structures and their variants (**A**) and the TMDs (**B**) throughout 1000 ns of MDS.

The WT-MRP4, G187W, and Y556C conformations during the first 100 ns of CG-MDS changed significantly, according to the RMSD values (Figure 4a), which indicates a large movement of the protein to further stabilization from 250 to 1000 ns. The RMSD of WT-MRP4 was higher than those of its variants, considering the complete structure. In addition, different regions of the MRP4 structure were studied focusing on the ligand binding sites, nsSNPs, and ATP pocket binding. Figure 4b shows the RMSD values for TMDs of the WT-MRP4 and its variants. According to the RMSD plot, the changes in the TMDs’ conformations are quite similar among the three MRP4 structures.

**Figure 5:**
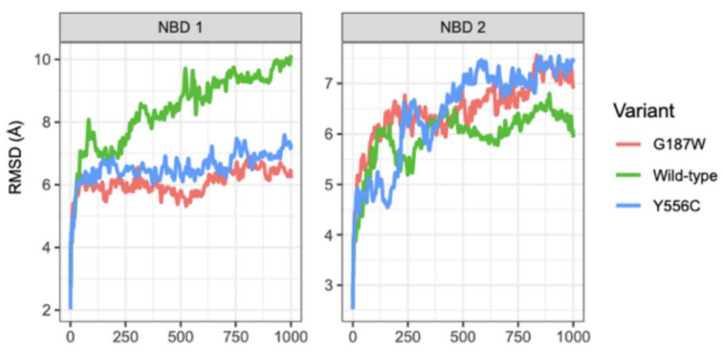
RMSD plot for NBD1 and NBD2 of WT-MRP4 and its variants throughout the 1000 ns of MDS.

In contrast to the complete structure, the TMDs do not obtain stabilization according to the RMSD values, which increase and decrease over 1000 ns. Moreover, the RMSD values for G187W remain increasing from 750 to 1000 ns, and such behavior could be due to the mutation is in the cytosolic loop 1 that connects the transmembrane helix (TMH) 1 and TMH2. The NBD1 conformation in WT-MRP4 remained unstable and the RMSD values kept increasing throughout the 1000 ns of CG-MDS, while the NBD1 conformation in G187W and Y556C was stable with an RMSD value around 6.0 Å (Figure 5). The RMSD values for NBD2 of the WT-MRP4 and its variants were similar even though there was no stabilization through the simulation. In addition, the RMSD values of G187W and Y556C tended to increase while the RMSD values of WT-MRP4 tended to decrease at the end of the simulation.

**Figure 6:**
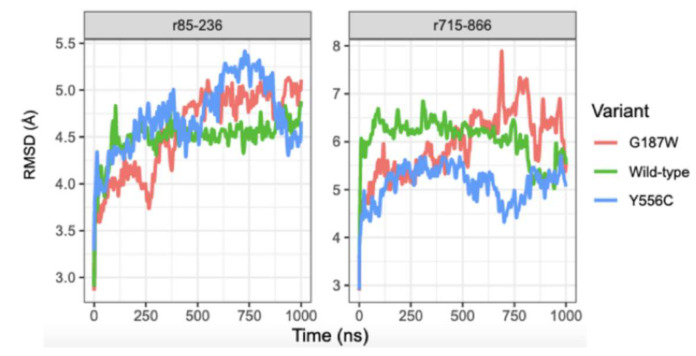
RMSD plot for r85-236 and r715-866 of WT-MRP4 and its variants. r86-236 represents TMH1-TMH4 and r715-866 represents TMH7-TMH10.

**Figure 7:**
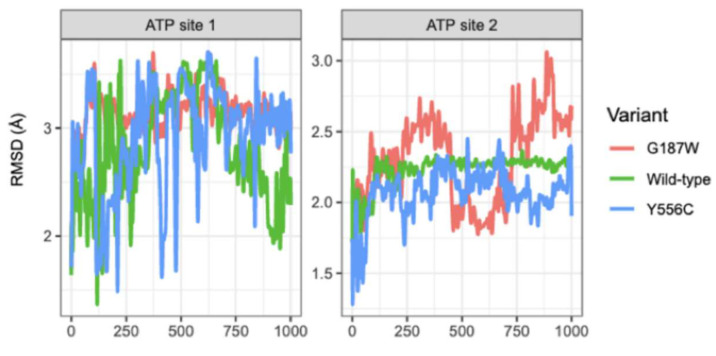
RMSD plot for ATP binding site 1 (ATP site 1) and ATP binding site 2 (ATP site 2) of WT-MRP4 and its variants.

According to the RMSD plot in Figure 6, the conformation in residues (r) 85 to 236 (r85-236 site corresponding to TMH1-TMH4) of WT-MRP4 remained stable from 250 to 1000 ns while in G187W it stabilized at the last 250 ns. r85-236 site in Y556C did not stabilize throughout the 1000 ns. In the same Figure 6, the RMSD plot indicates that the conformation in the r715-866 site (TMH7-TMH10) in WT-MRP4 and Y556C kept constant, with a tendency toward increasing motion in Y556C and decreasing motion in WT-MRP4. Besides this, the conformation in r715-866 site in G187W did not stabilize and the RMSD values suggest high motion throughout the 1000 ns of simulation. In the case of ATP sites 1 and 2 (Figure 7), the conformation remained unstable, suggesting that it is a site with high motion, with exception of ATP site 2 of WT-MRP4, which kept stable through the simulation. Regarding the three different MRP4 structures, alignments on the NBDs, TMDs, and TMH1-TMH4 and TMH7-TMH10 sites were performed to determine differences among the structures. In all the alignments (Supplementary Material S1), it was observed that all the sites studied presented significant structural differences, according to the RMSD values, comparing WT-MRP4 vs. its variants and G187W vs. Y566C. Moreover, the TMHs are responsible for the specificity for the substrate, and the r-85-236 and r715-866 sites in WT-MRP4 were significantly different with respect to G187W and Y556C, which could lead to differences in the ligand affinity, the ligand binding site, and the motion of the protein [23,24].

### 2.2. Molecular Docking in MRP4 and Variants

By using cluster 1 from CG-MDS for each structure of MRP4, molecular docking was performed to explore the effect of the MRP4 variants on the affinity of three different groups of molecules, previously reported as substrates or inhibitors in vitro. Table 1 presents the docking score (DS), expressed as kcal/mol, related to the interaction between endogenous substrates and WT-MRP4, Y556C, and G187W. In this work, significant differences between docking poses were considered when a difference greater than 1 kcal/mol in DS was present. According to the DS, most endogen substrates significantly changed their DS in G187W and Y556C with respect to WT-MRP4. The Y556C mutation is located at NBD1 and leads to a different conformation with respect to WT-MRP4, which causes the most substantial change in the ligand binding site of all the molecules studied, even more than those molecules with significant changes in DS compared to WT-MRP4.

Since cAMP is considered to be the main molecule transported by MRP4 [25], it was used as a control to compare the effect of nsSNPs and ATP binding. The cAMP DS was not considered significantly different in WT-MRP4 with respect to Y556C and G187W. Cholic acid DS was significantly different, with more than 3 kcal/mol when comparing WT-MRP4 with respect to G187W, while cholic acid DS in WT-MRP4 with respect to Y556C was significantly different at over 2 kcal/mol. The taurocholic acid DS was significantly different, with more than 4 kcal/mol when comparing WT-MRP4 with respect to G187W. Folic acid (FA) was the molecule with the highest variation in DS when comparing WT-MRP4 to G187W and Y556C, with 7.05 and 6.17 kcal/mol differences.

Table 2 presents the DSs of drug substrates. Cefazoline and olmesartan DSs were significantly different in WT-MRP4 with respect to G187W, while cefazoline, furosemide, leucovorin, methotrexate, tenofovir, and topotecan DSs were significantly different in WT-MRP4 with respect to Y556C. According to DS, most drug substrates could present more affinity for Y556C than for WT-MRP4.

The DS of inhibitors is presented in Table 3. Glafenine DS was significantly different in WT-MRP4 with respect to both G187W and Y556C. Ceefourin-1, indomethacin, and sildenafil DSs were significantly different in WT-MRP4 with respect to G187W, while losartan DS was significantly different between WT-MRP4 and Y556C. Endogen substrates were the group of molecules with more variation in the DS, and thus such mutations on the ABCC4 gene could lead to changes in cell metabolism related to changes in the distribution of endogen substrates across cell membranes. Several mutational studies on MRP1 have demonstrated that amino acids in several TMDs are involved in substrate binding and nsSNPs can modify the ligand binding site or the affinity of ligands in a selective manner [11]. Yet, the DS values are not totally related to function. Regarding drug substrates and inhibitors, a small number of molecules changed their DS significantly to G187W and Y556C as related to WT-MRP4, which could modify the pattern of transport of cefazoline, ceftizoxime, olmesartan, topotecan, and ceefourin-1, which could, possibly, mean that they act as substrates or inhibitors depending on the MRP4 variant.

### 2.3. Differences in the Interaction Pattern in MRP4 Structures

**Figure 8:**
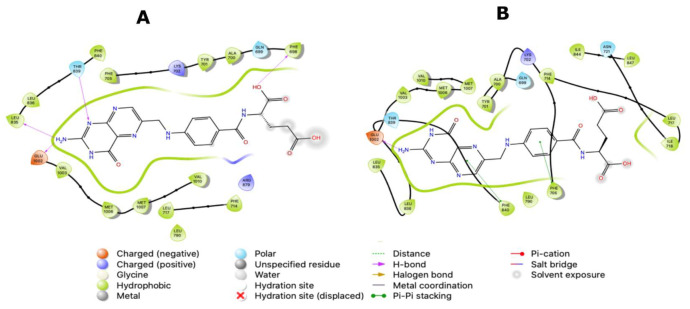
FA binding interaction diagram in WT-MRP4 at T0 (**A**) and 16.20 ns (**B**) in AA-MDS. The nomenclature for the intermolecular interactions is shown in the bottom of the figure.

**Figure 9:**
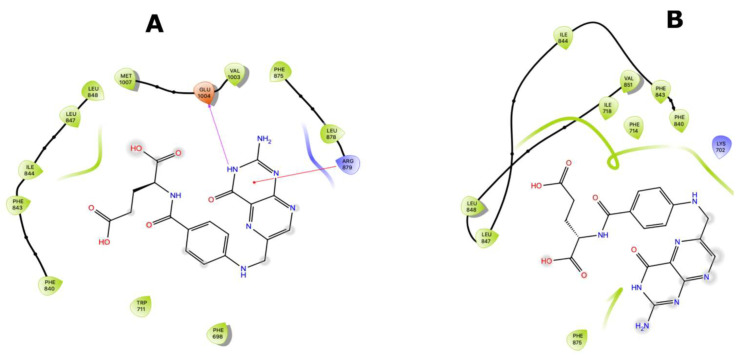
FA binding diagram interactions on G187W at T0 (**A**) and at 8.0 ns (**B**) in AA-MDS.

**Figure 10:**
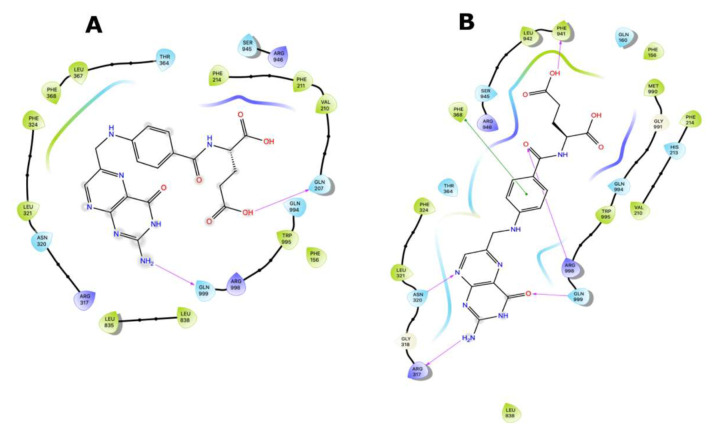
FA binding interaction diagrams in Y556C at T0 (**A**) and 15.0 ns (**B**) in AA-MDS.

Ligand interaction diagrams (LIDs) represent the pattern of intermolecular interactions of molecules with MRP4 amino acids. Those molecules with a >2 kcal/mol difference in the three different MRP4 structures appear in LIDs in Supplementary Material S2–S5 and the LIDs for FA appear in Figure 8, Figure 9 and Figure 10. The interaction sites in the three different MRP4 structures were different, mainly in Y556C, for all the molecules exhibited in the LIDs. Cholic acid interacts mainly with hydrophobic residues on WT-MRP4 and Y556C, while on G187W it interacts with hydrophobic and polar residues. H-bonds are only exerted through hydrophobic residues, except on Y556C, where arginine exerts an H-bond with cholic acid. In addition, cholic acid interacts with positively charged amino acids in the three MRP4 structures, lysine on G187W and WT-MRP4, and arginine on Y556C (Supplementary Material S2). The taurocholic acid binding site was different in WT-MRP4 with respect to G187W and was totally different with respect to Y556C. The intermolecular interactions in Y556C and G187W were H-bonds, polar, hydrophobic, interactions with positively and negatively charged amino acids, while interactions in WT-MRP4 were hydrophobic and polar but did not interact with negatively charged amino acids and did not exert H-bonds (Supplementary Material S3). The cefazoline binding site was quite different on the three different MRP4 structures. Hydrophobic and polar residues on WT-MRP4, Y556C, and G187W interact with cefazoline, but only on G187W do H-bonds with arginine occur. WT-MRP4 and Y556C interact with cefazoline through negatively charged amino acids (glutamate), while the G187W interacts through positively charged amino acids (arginine) (Supplementary Material S4). Ceefourin-1 interacts with hydrophobic, polar, negative, and positively charged residues on WT-MRP4 and Y556C, even though the binding site is different in each MRP4 structure, which leads to only one difference; ceefourin-1 interacts via H-bonds in Y556C. Additionally, ceefourin-1 interacts in G187W with hydrophobic, polar, and positively charged residues, and via H-bonds and π-π stacking (Supplementary Material S5). The Y556C mutation led to a substantial change in the binding site of most of the molecules analyzed, while G187W mutation did not substantially change the binding site with respect to WT-MRP4. Although the molecule’s binding site was different among the MRP4, the pattern of intermolecular interactions could be similar to that observed in the LIDs. The molecules presented in the LIDs had a substantial modification to their binding site and intermolecular interactions in Y556C, which could be related to changes in the transport-rate, IC50, entrance to the inner cavity, and effect on the Y556C conformational movement.

Table 4 shows the most important residues required for the interactions of endogen substrates, drug substrates, and inhibitors in each MRP4 structure. It is worth noting that in the inhibitor group, only Lys 329 and Arg 951 appear in both endogen and drug substrates, suggesting that such residues may play an important role in the binding site. In the endogen and drug substrate groups, at least two residues are repeated—Arg 946 and Lys 702.

### 2.4. All-Atom Molecular Dinaymics (AA-MD) Simulations and Umbrella Sampling Studies

FA was the molecule with the highest DS variation in G187W and Y556C related to WT-MRP4 in molecular docking studies, considering that ATP was not bound; hence, to study the effect of the presence of ATP and the mutations, changes in the pattern of intermolecular interactions and affinity to MRP4 structures, 25 ns AA-MDS, and 10 ns of Umbrella sampling simulations were carried out on the MRP4-FA complexes and compared with cAMP as a control molecule. The C1 in AA-MDS was at 16.2 ns in WT-MRP4, 8.0 ns in G187W, and 15.0 ns in Y556C. Figure 8 shows the FA LID in WT-MRP4 at T0 and 16.2 ns. In this simulation, the differences in the FA binding site and the pattern of intermolecular interaction can be observed according to the WT-MRP4 conformation at a given time. The FA binding site was the same at T0 and 16.2 ns, suggesting that the protein can be in an inward-facing conformation for 25 ns or even more. Moreover, the pattern of intermolecular interactions between FA and WT-MRP4 is quite similar, consisting of H-bonds; π-π stacking; and interactions with polar, hydrophobic, and negatively and positively charged residues. The differences in the pattern of intermolecular interactions rely on the H-bonds, with 4 H-bonds at T0 with Phe698 and Leu835, Glu1002 and Thr839. Meanwhile, at 16.2 ns the interactions were mainly hydrophobic (π-π stacking) and there was one H-bond with Glu1002. Therefore, it seems that FA from 0 to 16.2 ns interacts with MRP4 to achieve its optimal bonding only. A longer simulation will help to determine all the FA binding sites across the WT-MRP4. Besides this, the AA-MDS studies were performed on each MRP4-FA complex to determine differences in the patterns of interactions and conformations in frames from 0 to 25 ns every 5 ns referred to T0 (Supplementary Material S6–S23). As mentioned above, FA did not change its conformation significantly in WT-MRP4 from 0 to 25 ns, while in G187W the conformation was significantly different at 20 and 25 ns, as the carboxyl group was responsible for the FA conformational changes and multiple intermolecular interactions, such as H-bonds, π-π, and π-cation. FA in G187W did not change its binding site throughout all the AA-MD simulation, but it kept moving throughout 25 ns to obtain a DS greater than that of the most stable conformation at 8 ns (Supplementary Material S12–S17). The G187W mutation is located at cytosolic loop 1, close to the entrance of the inner cavity [26], and leads to changes in the MRP4 conformation which could block or interfere with ligand binding or the entrance to its binding site. Notwithstanding this, it is not possible to determine with this study whether there is a relevant effect on the FA transport by G187W. Figure 9 shows the FA LID in G187W at T0 and 8.0 ns in AA-MDS. The FA binding site was slightly different, with intermolecular interactions by an H-bond and π-cation at T0, while at 8.0 no intermolecular interactions were observed, but the pocket binding was composed of hydrophobic amino acids in both time steps.

Figure 10 shows the FA LID in Y556C at T0 and 15.00 ns in AA-MDS. In this case, the FA binding site was the same, but the intermolecular interactions by H-bonds at 15.00 ns were higher than at T0, which seems to confer to FA a stronger binding over time. Y556C promoted the greatest change in the FA binding site with respect to WT-MRP4 and G187W, but throughout the 25 ns in AA-MDS the binding site did not change. On the contrary, the pattern of intermolecular interactions as well as the FA conformation was different across the AA-MDS (Supplementary Material S18–S23).

**Figure 11:**
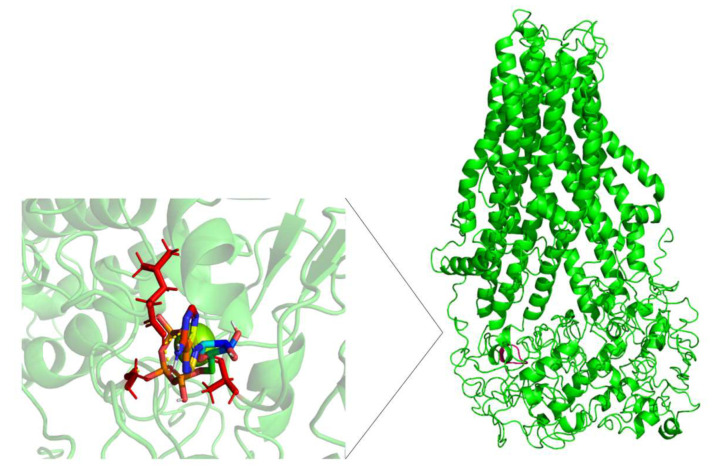
Representation of the ATP binding site 1 in the complete MRP4 structure and an amplification of such site. Color pink in the complete MRP4 structure represents the ATP site 1. Color red and represent the residues interacting with ATP, and rainbow colors represent ATP. The yellow dotted lines indicate polar interactions.

**Figure 12:**
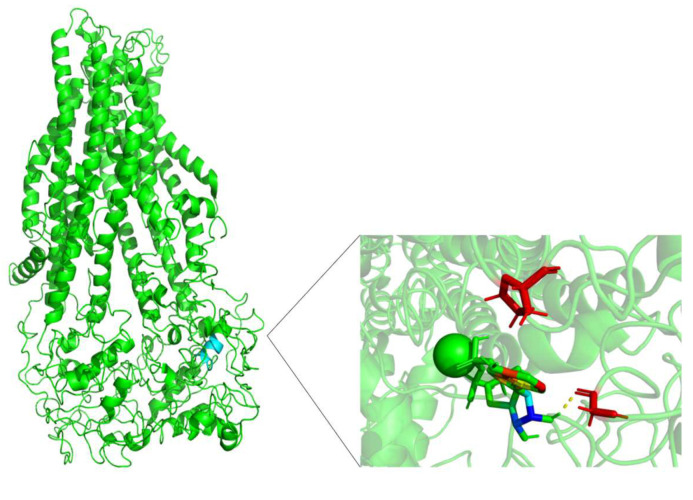
Representation of the ATP binding site 2 in the complete MRP4 structure and an amplification of such site. Color cyan in the complete MRP4 structure represents the ATP site 2. Color red represent the residues interacting with ATP, and rainbow colors represent ATP. The yellow dotted lines indicate polar interactions.

The AA-MDS studies were conducted in cAMP as a substrate control to determine if the nsSNPs affect the pattern of intermolecular interactions in the same manner as FA. First, AA-MDS were performed on the wild type and variants for MRP4-cAMP complex to obtain the C1 conformations. Further, ATP and Mg^2+^ were added into the MRP4 structures to obtain a more complete system, and further AA-MDS were performed to determine a new C1. This procedure was applied in the MRP4-FA complexes as well. Both C1 conformations before ATP and after ATP were used for umbrella sampling studies to obtain the free binding energy (ΔG) of each ligand. Since two ATP molecules bind to MRP4, we referred to them as ATP1 and ATP2 for the NBD1 and NBD2, respectively. In all the MRP4-FA and MRP4-cAMP complexes with ATP bonds, the binding site and the ligand conformation were slightly different concerning the MRP4-FA and MRP4-cAMP complexes without ATP. The presence or the absence of ATP modifies the pattern of intermolecular interactions as well. The H-bonds, π-π stacking, and π-cation interactions are present together or individually in all the complexes, except in G187W-FA without ATP. Given the fact that the ATP influences the ligand conformation, it modifies the intermolecular interactions as well. Figure 11 and Figure 12 show the ATP binding site in NBD1 and NBD2 from the WT-MRP4, G187W, and Y556C structures.

**Figure 13:**
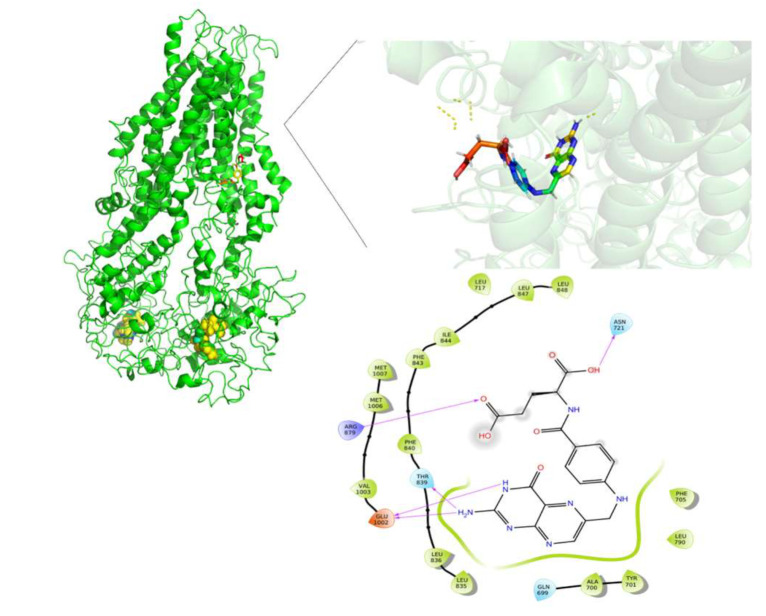
FA binding site in WT-MRP4 and amplification of such site. Rainbow colors in both the complete MRP4 structure and amplification of the binding site represent the FA structure while the yellow spheres and stick represent the ATP. The yellow dotted lines indicate polar interactions. The LID of WT-MRP4-FA is presented at the bottom.

**Figure 14:**
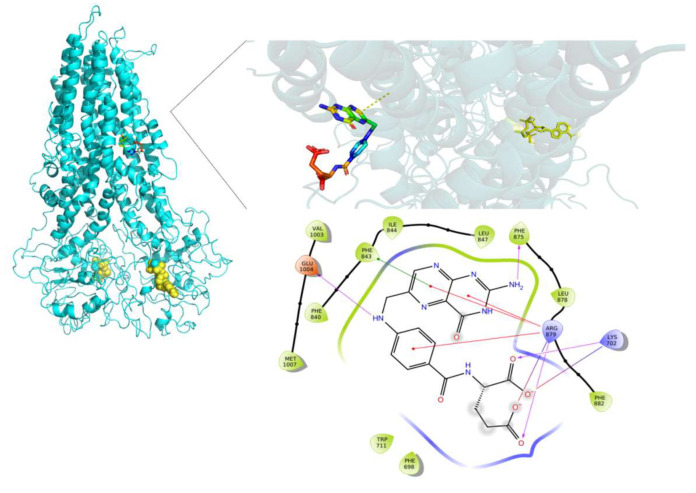
FA binding site in G187W and amplification of such site. Rainbow colors in both the complete WT-MRP4 and amplification of the binding site represent the FA structure while the yellow spheres and stick represent the ATP. The yellow dotted lines indicate polar interactions. The LID of G187W-FA is presented at the bottom.

**Figure 15:**
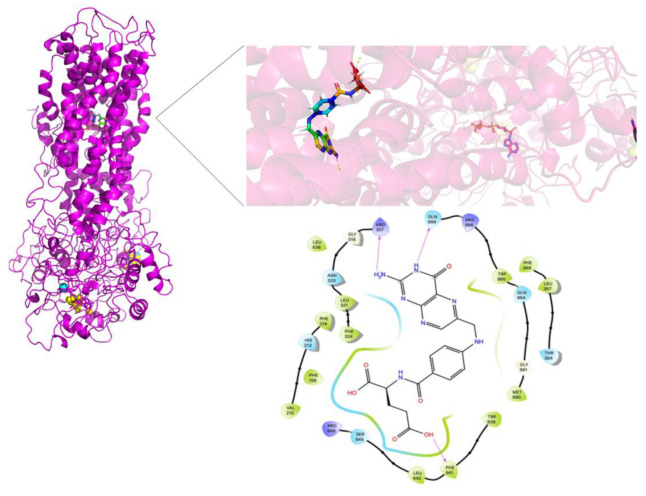
FA binding site in Y556C and amplification of such a site. Rainbow colors in both the complete G187W and amplification of the binding site represent the FA structure while yellow spheres represent the ATP. The yellow dotted lines indicate polar interactions. The LID of Y556C-FA is presented at the bottom.

Figure 13, Figure 14 and Figure 15 show the FA binding sites in WT-MRP4, G187W, and Y556C, respectively. All the afore mentioned figures contain the full MRP4 structure and a close-up of the ligand binding site. As observed in Figure 13 and Figure 14, the FA binding site is almost the same and it is surrounded by the TMH1-TMH3; meanwhile, Figure 15 shows that the FA binding site in Y556C is surrounded by TMH2-TMH7 deep in the inner cavity. This finding is interesting, due to the fact that the Y556C mutation could promote the greater affinity of FA for the Y556C variant by switching into a “high affinity inward-facing orientation”, making the binding of FA easier [27]. Nevertheless, this does not mean that the FA can be transported properly, since MRP4 has an altered conformation within the NBD2, which blocks the ATP hydrolysis but not ATP binding. However, MRP4 can function with the energy provided by one ATP hydrolysis, but the Y556C mutation could interfere with such ATP hydrolysis [28].

**Figure 16:**
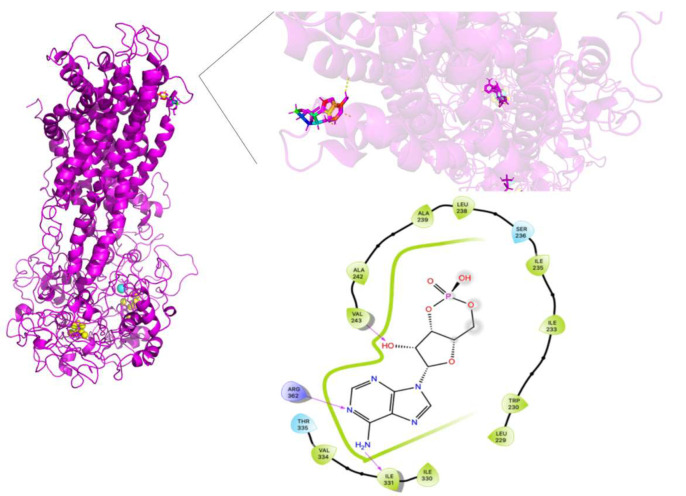
cAMP binding site in Y556C and amplification of such site. Rainbow colors in both the complete Y556C and amplification of the binding site represent the cAMP structure while yellow spheres represent the ATP. The yellow dotted lines indicate polar interactions. The LID of Y556C-cAMP is presented at the bottom.

Figure 16 shows that the cAMP binding site in Y556C is quite different compared to WT-MRP4 and G187W. The cAMP is located out of the normal binding site out of the TMDs and it may not be transported at a proper rate or efficacy. It seems that Y556C is in the “low-affinity outward-facing orientation”, where the main characteristic in this stage is that the affinity of the transported entity switches from high affinity (low chemical potential of the substrate) to low affinity (high chemical potential). Such change in affinity is due to the gate to the inside is closed, and the gate to the outside of the membrane is opened [23]. The global effect will depend on the cell type. Focusing on leukemia cells, could be harmful to them, because the intracellular cAMP levels would be increased and thus, leading to apoptosis [25]. On the other hand, the cAMP binding site in Y556C could represent the initial interaction only and further re-location such as the WT-MTP4. Longer AA-MDS are required to test this hypothesis.

**Figure 17:**
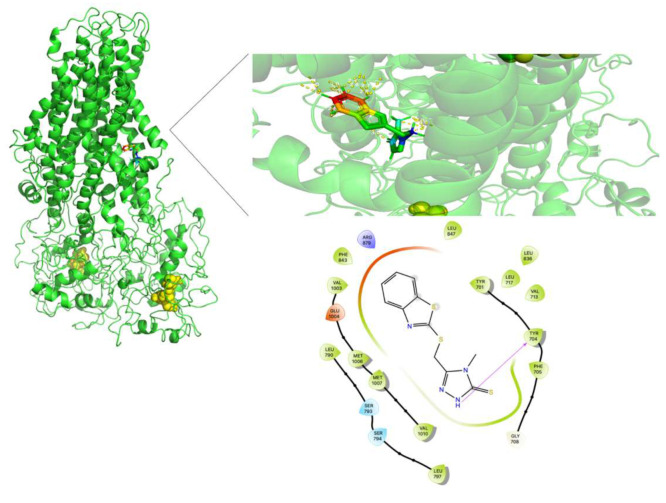
Ceefourin-1 binding site in WT-MRP4 and amplification of such site. Rainbow colors in both the complete WT-MRP4 and amplification of the binding site represent the ceefourin-1 structure while yellow spheres represent the ATP. The yellow dotted lines indicate polar and non-polar interactions. The LID of WT-MRP4-ceefourin-1 is presented at the bottom.

**Figure 18:**
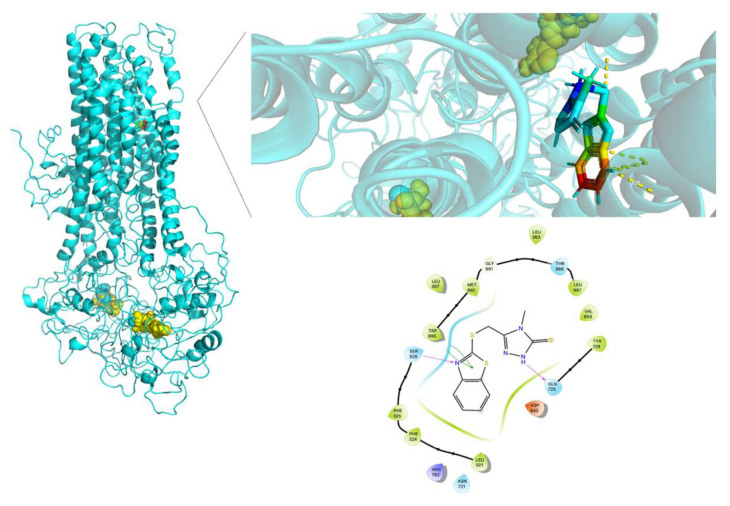
Ceefourin-1 binding site in G187W and the amplification of such a site. Rainbow colors in both the complete G187W and amplification of the binding site represent the ceefourin-1 structure, while yellow spheres represent the ATP. The yellow dotted lines indicate polar and non-polar interactions. The LID of G187W-ceefourin-1 is presented at the bottom.

**Figure 19:**
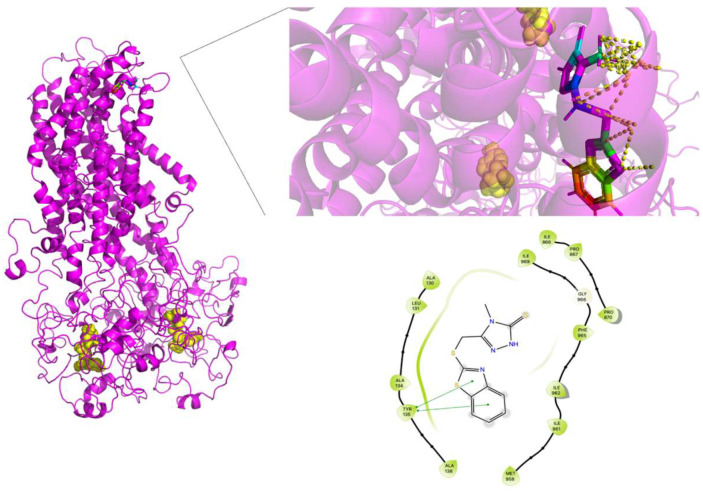
Ceefourin-1 binding site in Y556C and the amplification of such a site. Rainbow colors in both the complete Y556C and amplification of the binding site represent the ceefourin-1 structure, while yellow spheres represent the ATP. The yellow dotted lines indicate polar and non-polar interactions. The LID of Y556C-ceefourin-1 is presented at the bottom.

To contrast the effect of nsSNPs in MRP4, AA-MDS and umbrella sampling studies were performed in ceefourin-1, which is possesses a high selectivity over MRP4 inhibition. It has been suggested that ceefourin-1 may act as a competitive inhibitor in MRP4 [7], which is consistent to the AA-MDS where the binding site for cAMP and ceefourin-1 is similar in WT-MRP4, G187W and Y556C with ATP in the complex (Figure 17, Figure 18 and Figure 19). Interestingly, ceefourin-1 binds at the same abnormal binding site as cAMP in Y556C-ATP. Moreover, Y556C seems to be in the same “low-affinity outward-facing orientation” where the low affinity (high chemical potential) of the ligand leads to switch in the binding site [23]. As mentioned before, such abnormal binding site could be the initial binding site but longer AA-MDS is required to demonstrate it.

The cAMP and ceefourin-1 abnormal binding site in Y556C was one of the most remarkable findings in this work and showed the importance of studying the global effect of Y556C in different cell lines.

Umbrella sampling simulations were performed to determine the theoretical affinity, expressed as the free energy of binding (ΔG), of an event related to protein–ligand interactions along a reaction coordinate. Table 5 shows the ΔG obtained by Umbrella sampling through 10 ns of simulation on each complex. According to Umbrella sampling results, the Y556C-FA complex is the most stable with or without ATP bound to MRP4. Interestingly, ΔG for WT-FA and Y556C-FA complexes were lower in those MRP4 without ATP. In addition, ΔGs for G187W-cAMP and Y556C-cAMP complexes were higher without ATP, suggesting that the ATP bound is required for both FA and cAMP binding stabilization. ΔG for WT-MRP4-cAMP with or without ATP was the same. This represents an interesting finding because in WT-MRP4, the binding of ATP may promote the stabilization and conformational changes on MRP4 protein to promote an adequate interaction with FA to be properly transported out of the cell. Regarding the information provided by the LIDs, it is not possible to link the intermolecular interactions to ΔG because of the lack of a pattern in all the complexes. The intermolecular interactions do not define the increase or decrease of ΔG in this case. To confirm these observations, experimentally testing the FA efflux on cells expressing equal levels of WT-MRP4, G187W, and Y556C, as well as MRP4 inhibition by ceefourin-1 to measure the transport rate would afford further information of the differences among mutants and wild-type MRP4, experiments that are currently being carried out and will be reported in future articles.

The ΔG for the WT-MRP4-ATP1 complex was lower than that for FA and cAMP, which is reasonable considering its role in MRP4 functioning and needs to be bound to NBDs for longer time than ligands. In the case of ATP1/2 complexes with MRP4 variants, the most remarkable finding was that the Y556C-ATP1 complex was the least stable, theoretically demonstrated through the Umbrella sampling results, and it may be related to the mutation in the NBD1. The mechanism for the MRP4 function requires the transmission of the molecular motion from the NBDs to the TMDs. At this point, the ATP-binding can be considered as the power stroke in which the chemical potential of the transported entity changed, and ATP hydrolysis leads to the formation of an extra negative charge, thus opening the closed nucleotide sandwich structure and the opening of the nucleotide sandwich structure facilitates Pi release and ADP dissociation, which in turn allows the TMDs and access gates to reset to the high-affinity orientation on the original side of the membrane to continue the transport cycle [23,29]. If the Y556C mutation promotes a decreased affinity of ATP1 for its binding site and, in turn, a blockade of ATP1 hydrolysis, the Y556C variant activity could be diminished or truncated, also considering that WT-MTP4 and its variants lack the ability to hydrolyze ATP2 in NBD2; thus, the lack of Y556C efficacy in the transport substrates. Additionally, ATP2 had the highest affinity for Y556C, and it would be interesting to test if high affinity could reestablish the ATP2 hydrolysis in NBD2.

**Figure 20:**
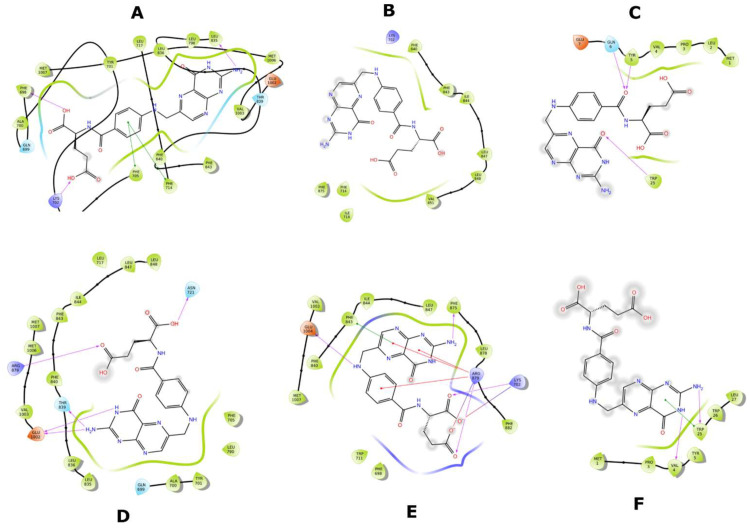
LIDs for FA with the different MRP4 structures with or without ATP. (**A**) WT-MRP4-FA complex. (**B**) G187W-FA complex. (**C**) Y556C-FA complex. (**D**) WT-MRP4-FA-ATP complex. (**E**) G187W-FA-ATP complex. (**F**) Y556C-FA-ATP complex.

**Figure 21:**
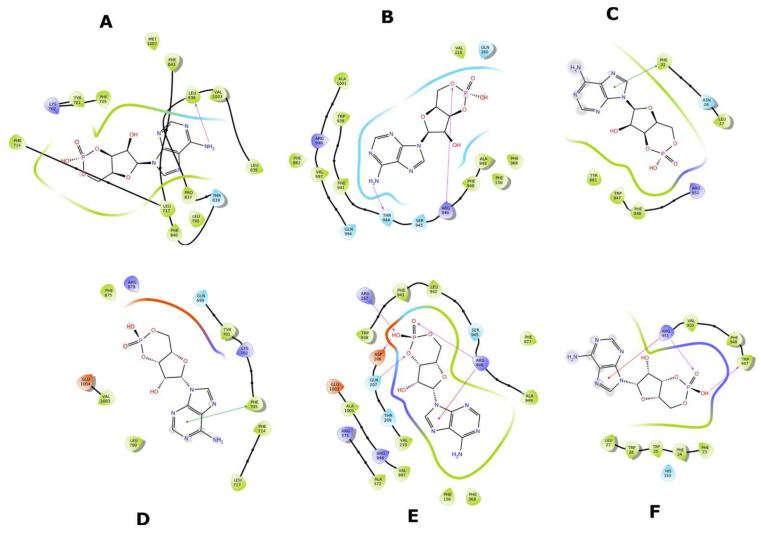
LIDs for cAMP with the different MRP4 structures with or without ATP. (**A**) WT-MRP4-cAMP complex. (**B**) G187W-cAMP complex. (**C**) Y556C-cAMP complex. (**D**) WT-MRP4-cAMP-ATP complex. (**E**) G187W-cAMP-ATP complex. (**F**) Y556C-cAMP-ATP complex.

**Figure 22:**
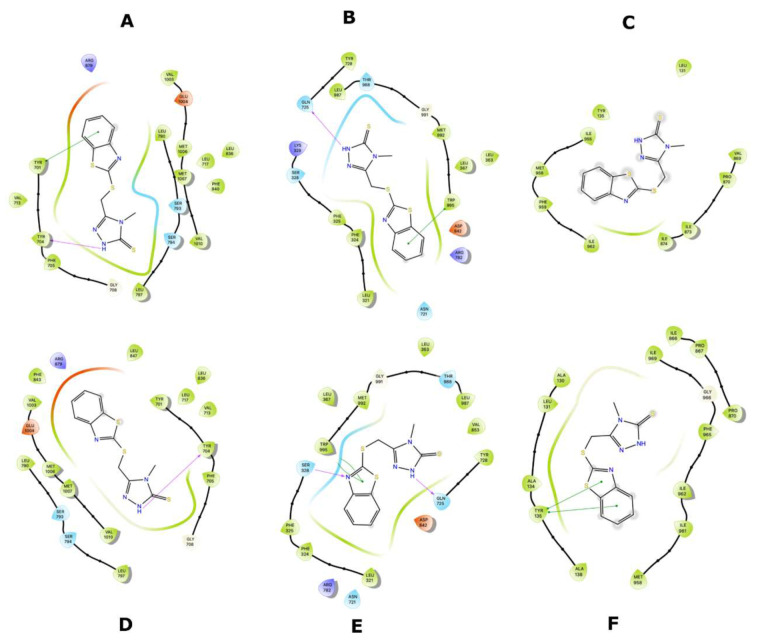
LIDs for ceefourin-1 with the different MRP4 structures with or without ATP. (**A**) WT-MRP4-ceefourin-1 complex. (**B**) G187W-ceefourin-1 complex. (**C**) Y556C-ceefourin-1 complex. (**D**) WT-MRP4-ceefourin-1-ATP complex. (**E**) G187W-ceefourin-1-ATP complex. (**F**) Y556C-ceefourin-1-ATP complex.

The abnormal cAMP and ceefourin-1 binding in Y556C (Figure 16 and Figure 19) is consistent with the ΔG for Y556C-cAMP and Y556-ceefourin-1, which had the lowest affinity with respect to the complexes with WT-MRP4 and G187W, and it indicates a low affinity at that binding site, suggesting two possibilities: (a) it is an initial cAMP or ceefourin-1 binding site to further binding at the inner cavity, or (b) possible deficiency in cAMP and ceefourin-1 transport. The latter is the most feasible due to cAMP interacts to G187W and WT-MRP4 in its normal binding site, but Y556C seems to be in the “low affinity outward-facing orientation” as mentioned before. To predict the effect of nsSNPs on the efficacy of chemotherapeutics, it is important to determine in a further study, the relation between ΔG in silico and the transport rate in vitro of several substrates. This suggests that ΔGs higher than that for the WT-cAMP-ATP complex may be related to a low rate of cAMP transport. The ΔGs for ceefourin-1 in all the complexes with MRP4 and its variants had the best affinity compared to those complexes of cAMP, supporting the idea that ceefourin-1 may act as a competitive inhibitor at least with cAMP. However, the presence of ATP in WT-MRP4 and its variants promotes a better affinity to cAMP than ceefourin-1 in the complexes with G187W and Y556C. A competitive assay in vitro is required to determine if ceefourin-1 had the best affinity to WT-MRP4 and its variants. The Figure 20 and Figure 21 represent the LIDs for FA and cAMP, respectively. In both figures, the intermolecular interactions of ligands with the absence of ATP are presented in the upper panel while the intermolecular interactions of ligands with the presence of ATP are shown in the bottom panel. The FA binding site is almost the same with or without ATP, but the number and the type of intermolecular interactions is different. It seems that ATP promotes a greater number of H-bonds between FA and all WT-MRP4 and its variants. Regarding the cAMP binding site, the ATP binding did not modify it; thus, the effect of ATP seems to be related with changes in the intermolecular interactions and affinity that, according to Umbrella sampling results, the ATP decreases the ΔG in the G187W-cAMP-ATP and Y556C-cAMP-ATP complexes. Figure 22 presents the LIDs for ceefourin-1. The binding site is different in all the complexes; interestingly, such a binding site does not change in the presence of ATP. The main difference induced for ATP is the intermolecular interaction pattern, which led to great amount of π-π stacking and H-bonds.

The MRP4 variants may predispose the population to a given disease regarding the site of the MRP4 that was affected by the mutation and the change in the affinity of a given substrate. The clinical implications of MRP4 variants have been studied over the past years and it is crucial to describe the relation of the MRP4 variants with diseases [30].

## 3. Materials and Methods

### 3.1. Protein Threading for WT-MRP4 and Its Variants G187W and Y556C

Structure prediction by protein threading for MRP4 was performed using its primary sequence (code: O15439) from the UniProt database [31,32]. MRP4 mutant models were made by the substitution of amino acids, G187W, or Y556C into the WT-MRP4 primary sequence. Each primary sequence was uploaded into the I-TASSER [17,18] server for the calculations of the models. The crystallography of MRP1 (PDBD code: 5UJ9) from *Bos taurus* was used as a template in all cases [33,34].

### 3.2. Coarse-Grained Molecular Dynamics (CG-MD) Simulations

All the systems (WT, G187W, and Y556C) for the simulations were built in the Martini Maker module [35] from CHARMM-GUI [36,37] using the Martini2.2p force field and adding a phosphatidylcholine (POPC) lipid bilayer membrane in an isothermal-isobaric ensemble (NPT) at 310.15 K. The simulations of 1 μs CG-MD were carried out in the Gromacs 2018.7 program [38,39], after of the minimizations and equilibrium protocols suggested by CHARM-GUI server. The module “cluster” of Gromacs 2018.7 was used to find the relevant conformations of the simulation using the “gromos” algorithm and the backbone for alignment. The most representative structure of the largest conformation cluster of the three simulations was converted into all atoms in the Martini Maker/All-Atom converter from CHARMM-GUI for the following calculations.

All the CG-MD simulations were performed in the ADA cluster of the National Laboratory of Advanced Scientific Visualization at Campus Juriquilla of the National Autonomous University of Mexico (LAVIS-UNAM).

### 3.3. Molecular Docking on WT-MRP4, G187W and Y556C

The 3D structures of the selected ligand groups, substrate drugs, endogenous substrates, and inhibitors were obtained from the PubChem public database [40]. Molecular docking was performed with AutoDock 4.2.6 optimized for graphical-processing units using a total of 50 runs and 25,000,000 evaluations; a grid box of 22.5 × 22.5 × 22.5 Å^3^ centering on the relevant amino acids reported by Ravna 2008 and 2009, El-Sheink 2008, and Chen 2018 [9,41,42,43]; in a Lamarckian genetic algorithm and Solis-Wets local search [44].

### 3.4. All-Atom Molecular Dynamics (AA-MD) Simulations

For molecular dynamic studies with FA as a ligand, the protein–ligand complexes WT-MRP4-FA, G187W-FA, or Y556C-FA, obtained from the molecular docking, were used, and AA-MD simulations were performed in Desmond 3.6 as an application of the Maestro software [45,46] as graphical interface. The AA-MDS systems were built using the System Setup module with an OPLS force-field (Optimized Potentials for Liquid Simulations), adding a POPC lipid membrane and simple point charge (SPC) water model in an NPT assembly at 310.15 K. Once the system was built, the standard relaxation protocol for system relaxation with increasing temperatures and decreasing restraints was used and an MD production simulation of 25 ns (100 frames) was performed in the Molecular Dynamics module. Clustering was performed in the Desmond Trajectory Clustering module to obtain the most representative conformation of the largest cluster (cluster1, C1). Trajectory analyses were performed in the Simulation Event Analysis module in Maestro.

### 3.5. Umbrella Sampling

Using the C1 of the 25 ns AA-MD trajectory, the system was built under the previously mentioned conditions. Once the system was obtained and relaxed using the AA-MD relaxation protocol, a 10 ns (100 frames) MD simulation was performed in the Metadynamics module of Desmond using the protein and ligand center-of-mass distance as the collective variable, with 0.3 kcal/mol height and 0.1 kcal/mol width as the Gaussian parameters for the umbrella protocol, on an NPT ensemble at 310.15 K and 1.01325 bar. Finally, the analysis was performed in the Metadynamics Analysis module of Desmond.

### 3.6. Manipulation of the Complexes and Figures

All the protein figures and alignments presented in this work were made in PyMOL software (The PyMOL Molecular Graphics System, Version 1.2r3pre, Schrödinger) [47]. The manipulation of the WT-MRP4, G187W, and Y556C structures was performed in Maestro (Schrödinger Release 2019-3: Maestro, Schrödinger, LLC, New York, NY, USA) [48].

## 4. Conclusions

To obtain the 3D structure of MRP4 and its variants, which are not resolved by NMR or X-ray, protein threading was performed in this study and relaxing the structures was performed by CG-MDS to carry out the molecular docking, while MDS and umbrella sampling studies were performed to yield relevant information regarding the residues involved in the binding of the studied molecules groups and changes in the ΔG of FA and cAMP in the presence or the absence of ATP, which also allowed us to observe the relevance of the mutations in the binding and movement of MRP4 and its variants. According to our results, the nsSNPs G187W and Y556C led to changes in the ligand binding site, DS, and binding energy (ΔG). In addition, the ATP binding to MRP4 significantly modifies the intermolecular interactions (at least, for FA and cAMP) and the binding energy compared to the complexes where ATP was not bound to MRP4. The effect of the abnormal binding site of cAMP in Y556C is consistent with the highly selective MRP4 inhibitor ceefourin-1, which makes it interesting to study such mutations in vitro. The affinity of ceefourin-1 for WT-MRP4 and its variants is higher than the affinity of cAMP. Cofactors such as ATP and Mg^2+^ should be included in the in silico analyses related to MRP4. It is well known that non-synonymous mutations usually affect the protein function or activity and its conformation, but this is the first report that suggests that most endogen substrates change their affinity and binding site in G187W and Y556C, which could modify the cell metabolism. We will report in further works the measure of substrate efflux and the relation between G187W or Y556C expression, location, and cell viability to determine the overall effect of the nsSNPs G187W and Y556C in vitro.

## Figures and Tables

**Table 1:** Endogen substrate DS (kcal/mol) in the WT-MRP4 and MRP4 variants.

Endogen Substrate	WT-MRP4	G187W	Y556C
cAMP	−5.88	−6.35	−5.82
Cholic acid	−10.55	−7.2 *	−8.51 *
Folic acid	−8.56	−1.51 *	−2.39 *
Glycolic acid	−7.35	−9.09 *	−5.42 *
Leukotriene B4	−8.19	−7.82	−6.07 *
PGE1	−8.21	−7.31	−7.48
PGE2	−7.36	−7.31	−7.00
Prasterone sulphate	−10.65	−9.24 *	−9.57 *
Taurocholic acid	−4.61	−9.9 *	−6.07 *
Uric acid	−4.77	−5.13	−5.06

cAMP: cyclic adenosine monophosphate. * Represents more than 1 kcal/mol of difference in the DS compared with WT.

**Table 2:** Drug substrate DS (kcal/mol) in the WT-MRP4 and MRP4 variants.

Drug Substrate	WT-MRP4	G187W	Y556C
6-Mercaptopurine	−4.34	−5.06	−4.81
Adefovir	−3.54	−2.88	−4.42
Cefazoline	−8.71	−5.65 *	−10.12 *
Cefotaxime	−7.04	−6.08	−6.88
Ceftizoxime	−7.02	−7.16	−6.59
Furosemide	−5.77	−5.52	−7.00 *
Hydrochlorothiazide	−6.70	−6.39	−6.73
Leucovorin	−6.46	−6.60	−3.78 *
Methotrexate	−6.98	−7.22	−3.76 *
Olmesartan	−7.30	−8.75 *	−7.4
Tenofovir	−2.76	−3.15	−4.58 *
Topotecan	−6.09	−6.39	−8.26 *

* Represents more than 1 kcal/mol difference in the DS compared against WT.

**Table 3:** Drug inhibitor DS (kcal/mol) in the WT-MRP4 and MRP4 variants.

Inhibitors	WT-MRP4	G187W	Y556C
ABSF	–5.41	–5.85	–5.97
Artesunate	–6.02	–5.42	–6.59
Ceefourin1	–7.52	–6.19 *	–7.22
Celecoxib	–7.57	–8.16	–7.98
Dipyridamole	–3.86	–4.78	–4.23
Glafenine	–8.27	–5.48 *	–6.32 *
Indomethacin	–8.42	–7.07 *	–8.43
Losartan	–8.82	–8.91	–6.55 *
MK-571	–8.86	–9.65	–8.87
Parthenolide	–7.88	–6.92	–7.67
Prazosin	–6.71	–6.98	–7.25
Probenecid	–6.59	–5.89	–6.34
Quercetin	–6.16	–6.2	–6.63
Sildenafil	–7.67	–8.43 *	–8.25
Sulindac	–8.41	–7.82	–8.41
Tyrphostin	–7.74	–7.32	–8.11

* Represents more than 1 kcal/mol difference in the DS compared against WT.

**Table 4:** Residues considered as important for ligand–MRP4 interactions.

	WT-MRP4	G187W	Y556C
Endogen substrates	Glu 1002, Lys 702, Thr 839, Lys 106, Lys 329, Glu 374, Gln 251, Phe 698	Arg 946, Arg 998. Gly 991, Lys 702, Arg 946	Arg 951, Trp 947, Glu 7, Gln 6, Val4
Drug substrates	Arg 946, Arg 998, Lys 702, arg 946, Thr 994, arg 312, Lys 329, Phe 698	Ser 945, Lys 329, Leu 987, Arg 946, Arg 951, Arg 312, Ser 306	Arg 946, Asp 15, Phe 329, Lys 32, Phe 939
Inhibitors	Phe 993, Pro 867, Arg 951, Arg 362, Thr 366, Gln 251, Leu 247	Phe 325, Lys 329, Thr 364, Glu 102, Glu 103, Trp 995	Phe 993, Pro 867, Arg 951, Trp 947

**Table 5:** Binding free energies (ΔGs) obtained by umbrella sampling.

	ΔG (kcal/mol)		
Ligand	WT-MRP4	G187W	Y556C
FA	–25.340	–23.288	–46.4898
FA-ATP	−16.490	−23.429	−38.9452
FA-ATP1	−46.010	−29.343	−18.7461
FA-ATP2	−37.226	−18.690	−61.100
cAMP	−17.700	−31.014	−12.5676
cAMP-ATP	−17.721	−39.300	−31.560
cAMP-ATP1	−43.966	−33.690	−49.7395
cAMP-ATP2	−23.860	−47.230	−38.5368
Ceefourin-1	−25.9938	−33.8589	−18.8326
Ceefourin-1-ATP	−22.9107	−19.7664	−22.5503
Ceefourin-1-ATP1	−40.9182	−43.1199	−43.1179
Ceefourin-1-ATP2	−37.2706	−52.9313	−53.6592

FA-ATP, cAMP-ATP, and ceefourin-1-ATP: the ΔG is referred to FA, cAMP or ceefourin-1 in complex with ATP. FA-ATP1, FA-ATP2, cAMP-ATP1, cAMP-ATP2, ceefourin-1-ATP1, and ceefourin-1-ATP2: ΔG calculation is focused on ATP1 or ATP2.

## Supplementary Materials

The following are available online: Table S1: RMSD values obtained for the alignment of C1 cluster from AA-MDS for different sites of WT-MRP4, G187W and Y556C, Figure S2: Binding site and intermolecular interaction of cholic acid in WT-MRP4 (A), G187W (B) and Y556C (C). D Nomenclature of LIDs, Figure S3: Binding site and intermolecular interaction of taurocholic acid in WT-MRP4 (A), G187W (B) and Y556C (C), Figure S4: Binding site and intermolecular interactions of cefazoline in WT-MRP4 (A), G187W (B) and Y556C (C), Figure S5: Binding site and intermolecular interaction of ceefourin-1 in WT-MRP4 (A), G187W (B) and Y556C (C), Figure S6: WT-MRP4-FA complex at T0 in AA-MDS, Figure S7: WT-MRP4-FA complex at 5 ns in AA-MDS, Figure S8: WT-MRP4-FA complex at 10 ns in AA-MDS, Figure S9: WT-MRP4-FA complex at 15 ns in AA-MDS, Figure S10: WT-MRP4-FA complex at 20 ns in AA-MDS, Figure S11: WT-MRP4-FA complex at 25 ns in AA-MDS, Figure S12: G187W-FA complex at T0 in AA-MDS, Figure S13: G187W-FA complex at 5 ns in AA-MDS, Figure S14: G187W-FA complex at 10 ns in AA-MDS, Figure S15: G187W-FA complex at 15 ns in AA-MDS, Figure S16: G187W-FA complex at 20 ns in AA-MDS, Figure S17: G187W-FA complex at 25 ns in AA-MDS, Figure S18: Y556C-FA complex at T0 in AA-MDS, Figure S19: Y556C-FA complex at 5 ns in AA-MDS, Figure S20: Y556C-FA complex at 10 ns in AA-MDS, Figure S21: Y556C-FA complex at 15 ns in AA-MDS, Figure S22: Y556C-FA complex at 20 ns in AA-MDS, Figure S23: Y556C-FA complex at 25 ns in AA-MDS.
