# Supplementary material for: Study of Endogen Substrates, Drug Substrates and Inhibitors Binding Conformations on MRP4 and Its Variants by Molecular Docking and Molecular Dynamics
Source: Molecules. 2021 Feb 17;26(4):1051. doi: 10.3390/molecules26041051 (PMC7922701; doi:10.3390/molecules26041051)
Supplement: Supplementary file 1 [file molecules-26-01051-s001.pdf]

**Supplementary material**

S1. RMSD values obtained for the alignment of C1 cluster from AA-MDS for different sites of WT-MRP4, G187W and Y556C

| Alignment | RMSD value | Significant or not significant |
|---|---|---|
| WT-MRP4 (TMDs)/G187W (TMDs) | 3.927 Å | Significant |
| WT-MRP4 (TMDs)/Y556C (TMDs) | 4.139 Å | Significant |
| G187W (TMDs)/ Y556C (TMDs) | 4.485 Å | Significant |
| WT-MRP4 (r85-248)/ G187W (r85-248) | 3.323 Å | Significant |
| WT-MRP4 (r85-248)/ Y556C (r85-248) | 3.801 Å | Significant |
| G187W (r85-248)/ Y556C (r85-248) | 5.027 Å | Significant |
| WT-MRP4 (r715-866)/ G187W(r715-866) | 4.485 Å | Significant |
| WT-MRP4 (r715-866)/ Y556C (r715-866) | 4.555 Å | Significant |
| G187W (r715-866)/ Y556C (r715-866) | 4.528 Å | Significant |

Ligand binding diagram interactions

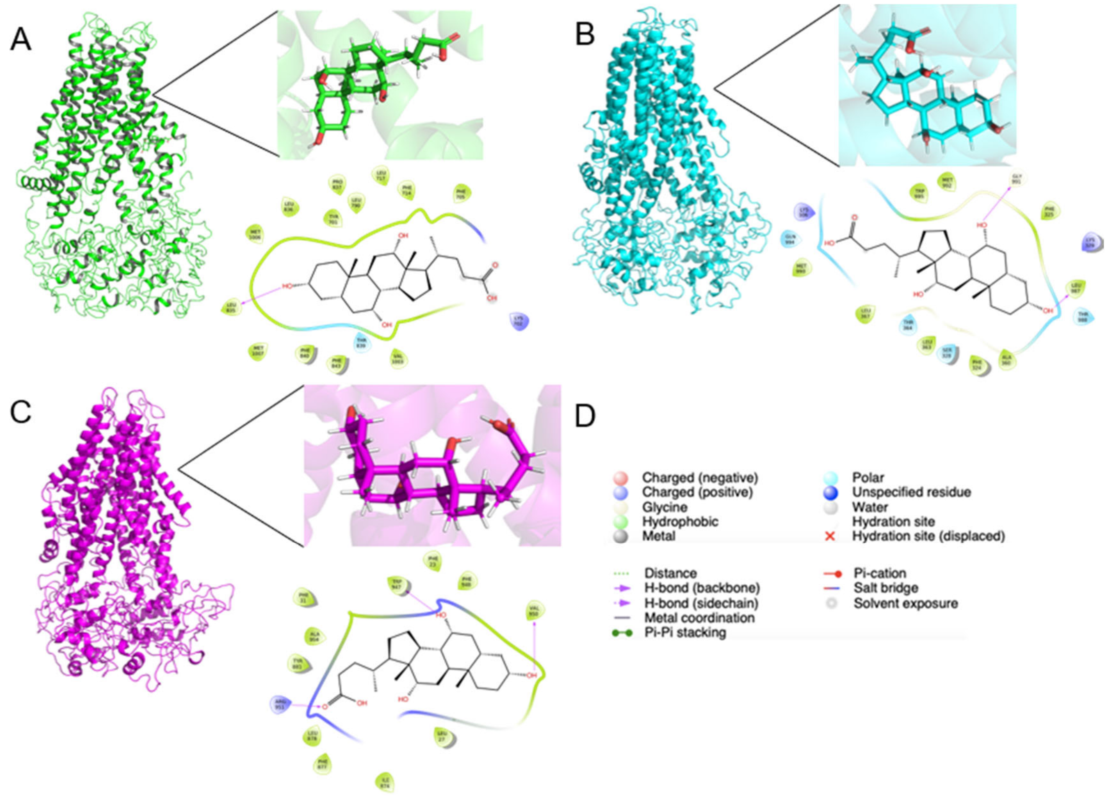


S2. Binding site and intermolecular interaction of cholic acid in WT-MRP4 (**A**), G187W (**B**) and Y556C (**C**). **D** Nomenclature of LIDs.

***Nomenclature for the LIDs is presented in S2 only.**

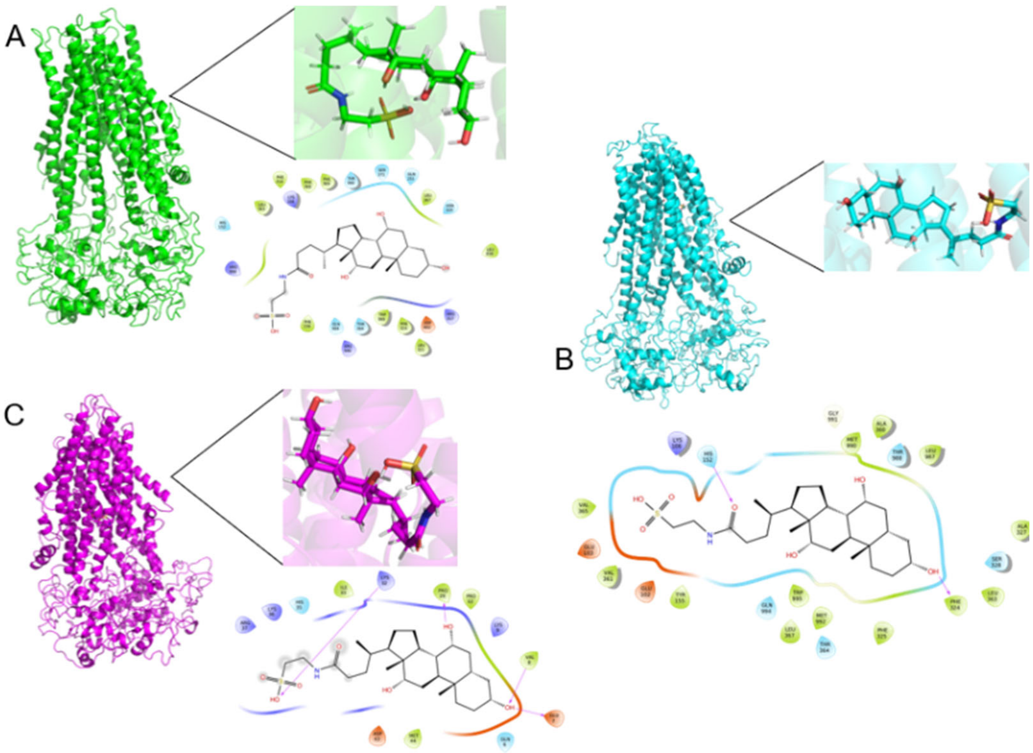


**S3.** Binding site and intermolecular interaction of taurocholic acid in WT-MRP4 (**A**), G187W (**B**) and Y556C (**C**).

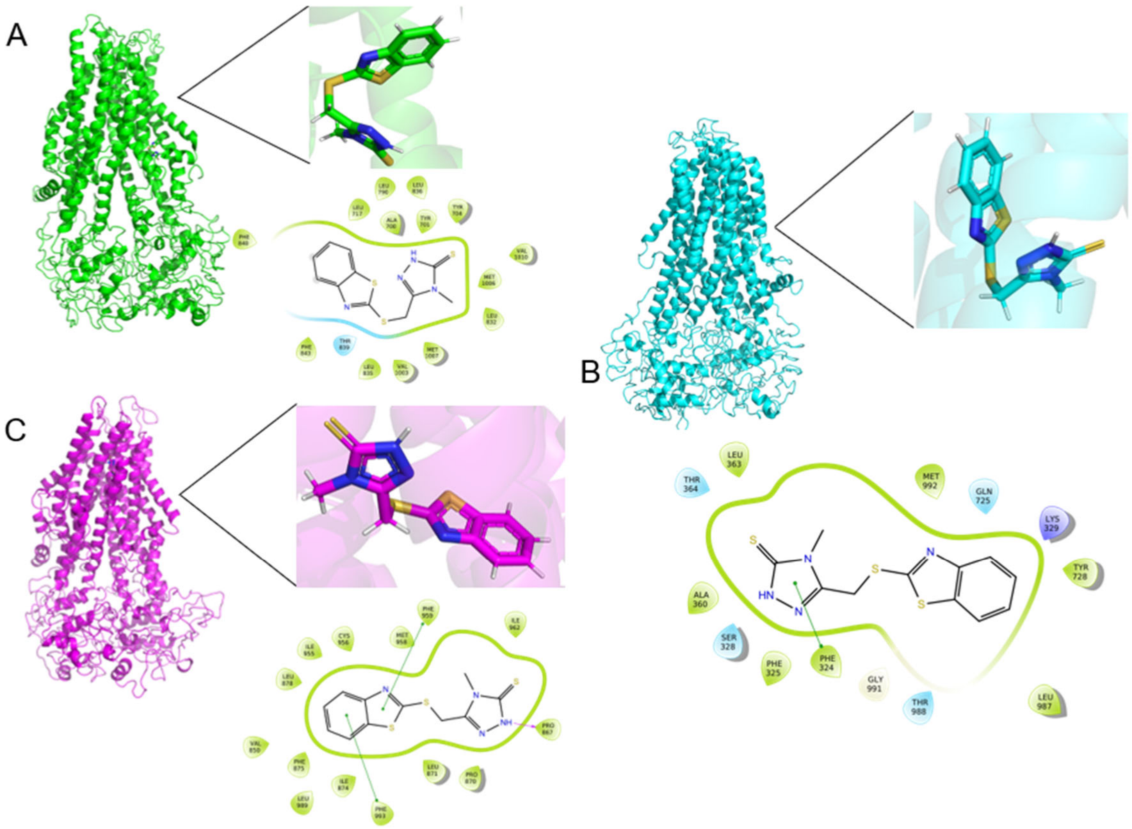


S4. Binding site and intermolecular interactions of cefazoline in WT-MRP4 (**A**), G187W (**B**) and Y556C (**C**).

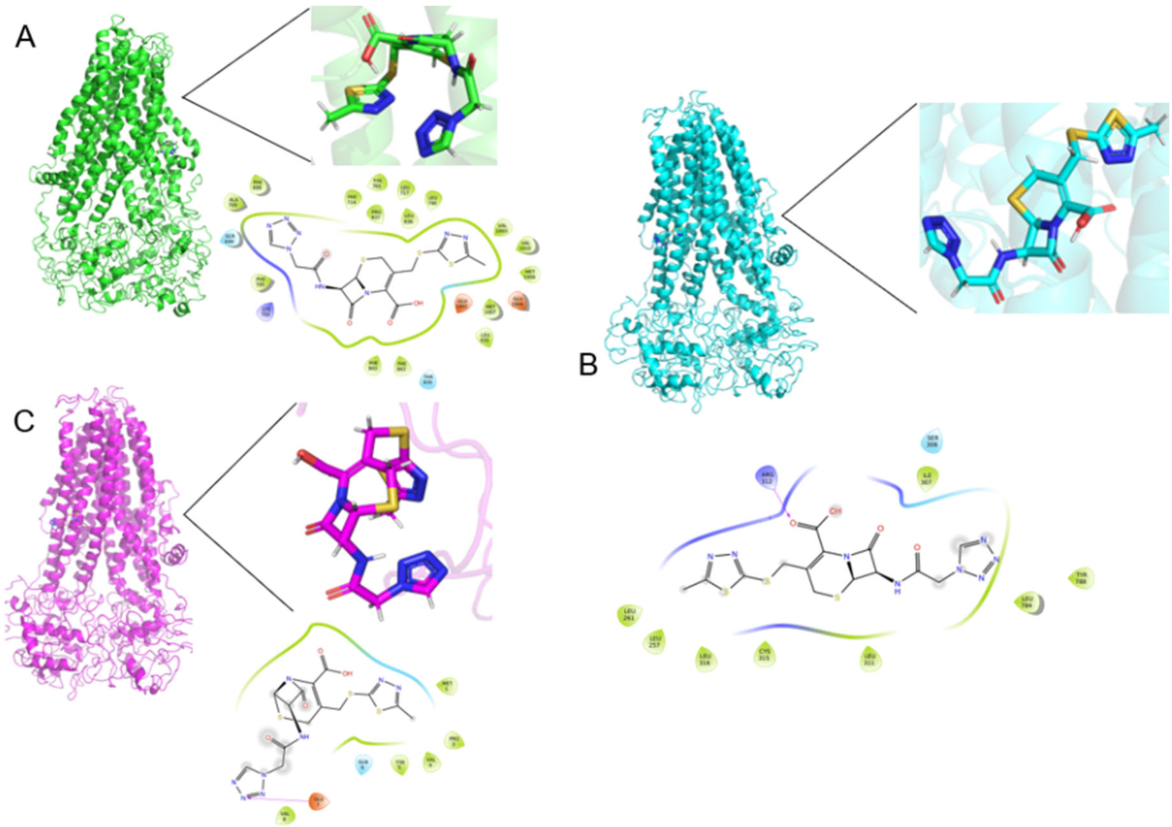


S5. Binding site and intermolecular interaction of ceefourin-1 in WT-MRP4 (**A**), G187W (**B**) and Y556C (**C**).

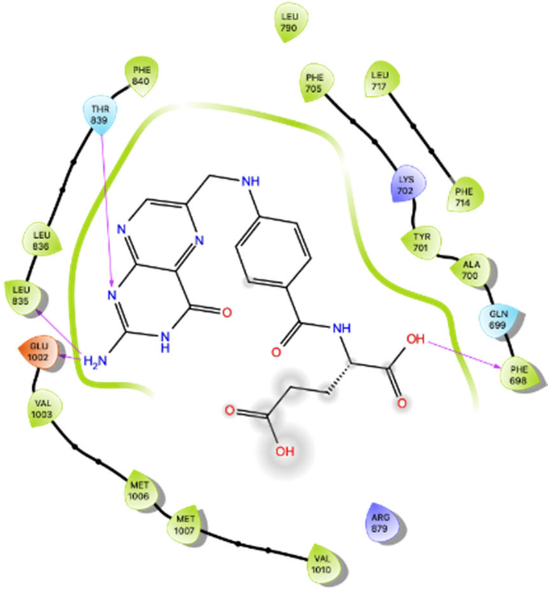


S6. WT-MRP4-FA complex at T0 in AA-MDS

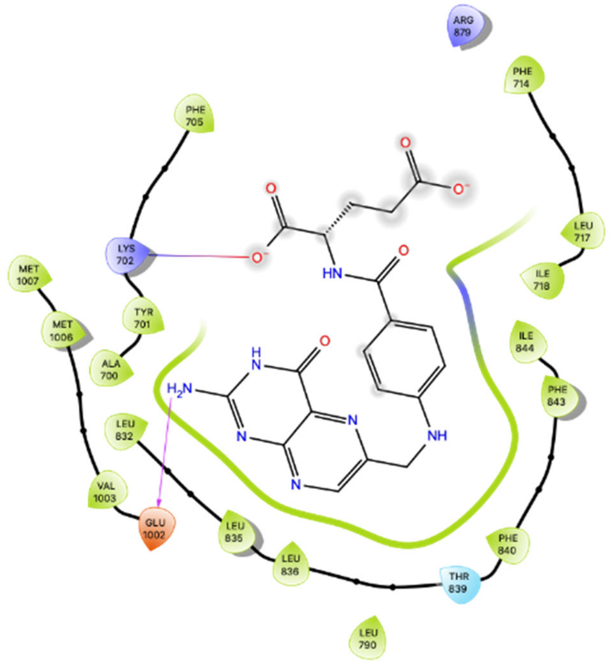


S7. WT-MRP4-FA complex at 5 ns in AA-MDS

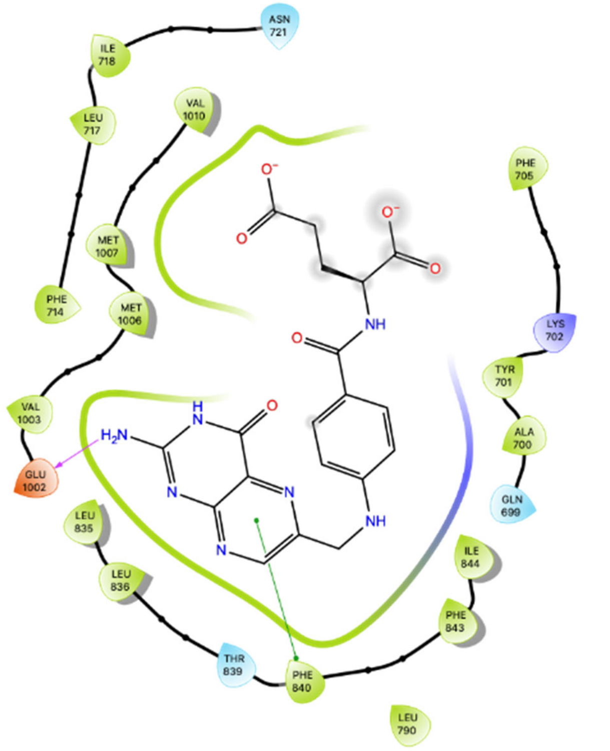


S8. WT-MRP4-FA complex at 10 ns in AA-MDS

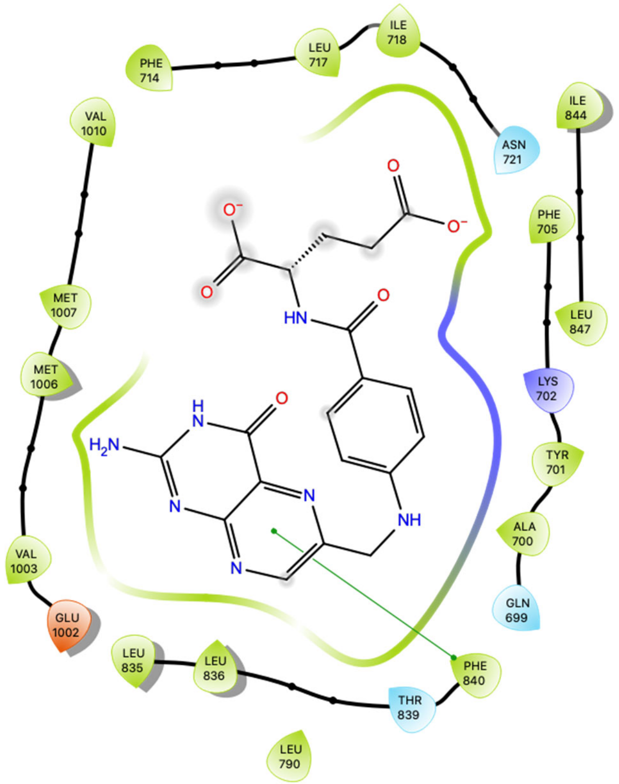


S9. WT-MRP4-FA complex at 15 ns in AA-MDS

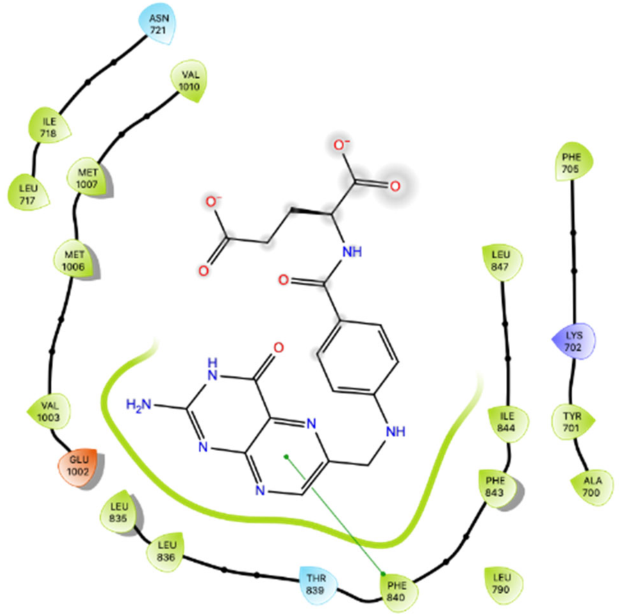


S10. WT-MRP4-FA complex at 20 ns in AA-MDS

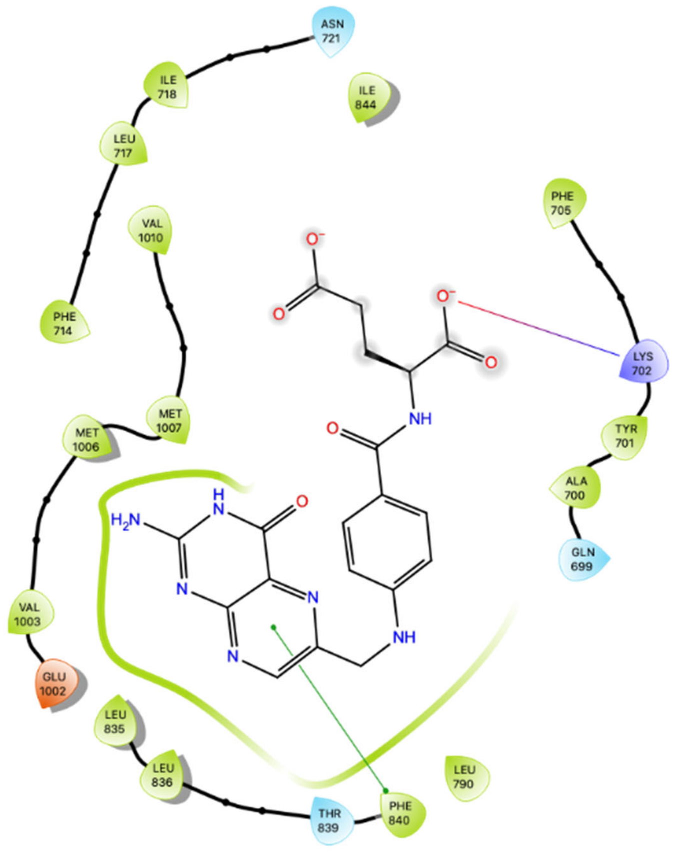


S11. WT-MRP4-FA complex at 25 ns in AA-MDS

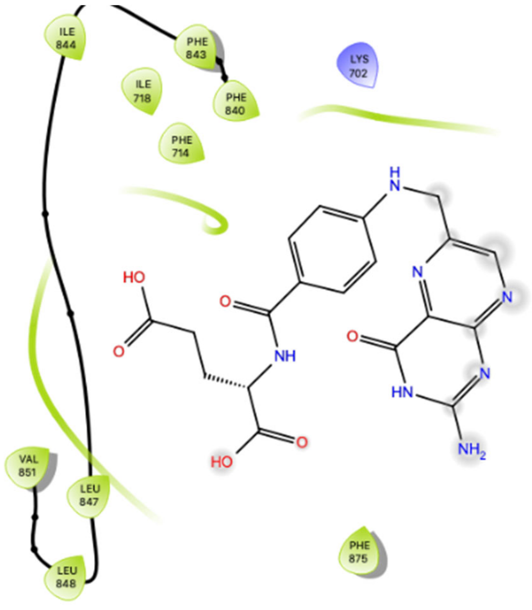


S12. G187W-FA complex at T0 in AA-MDS

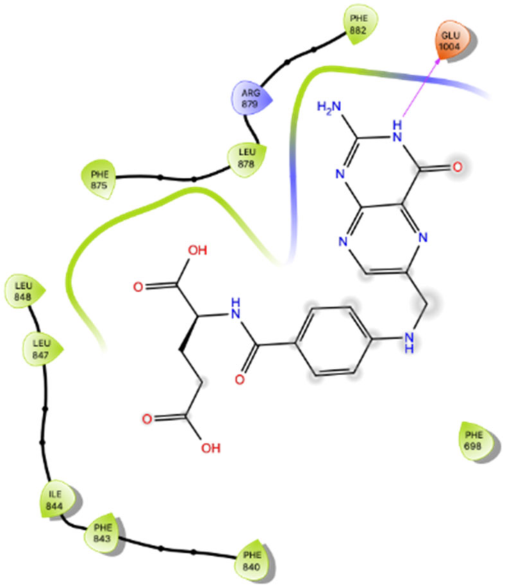


S13. G187W-FA complex at 5 ns in AA-MDS

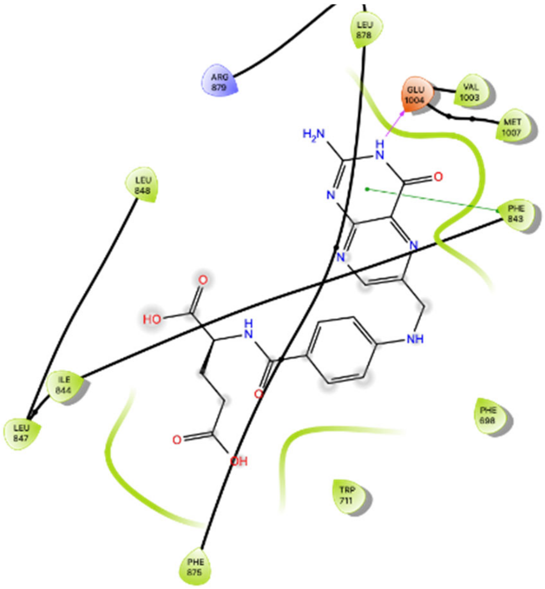


S14. G187W-FA complex at 10 ns in AA-MDS

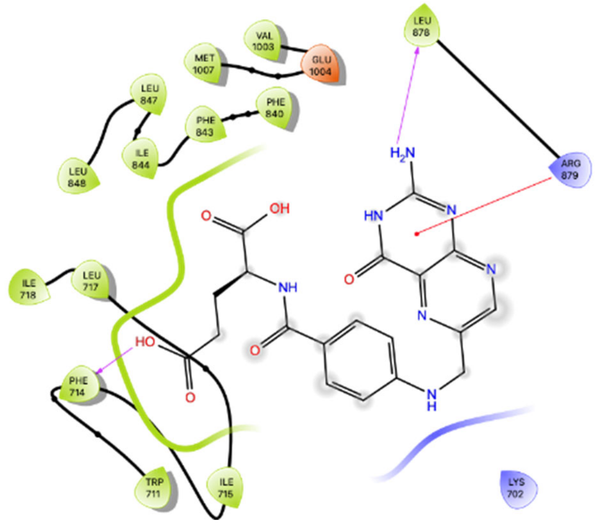


S15. G187W-FA complex at 15 ns in AA-MDS

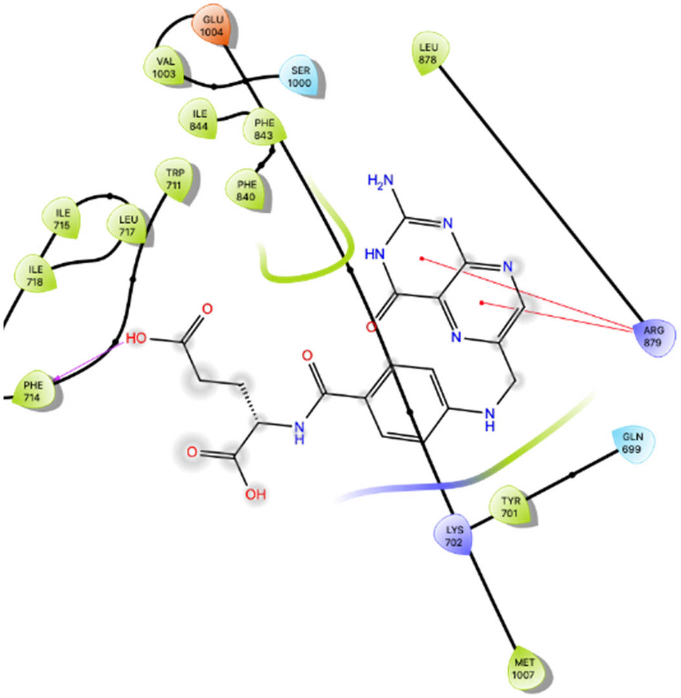


S16. G187W-FA complex at 20 ns in AA-MDS

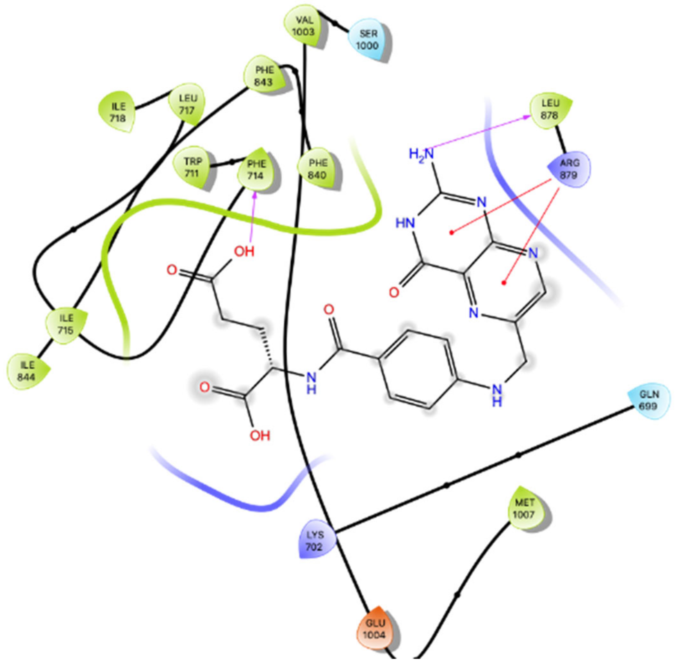


S17. G187W-FA complex at 25 ns in AA-MDS

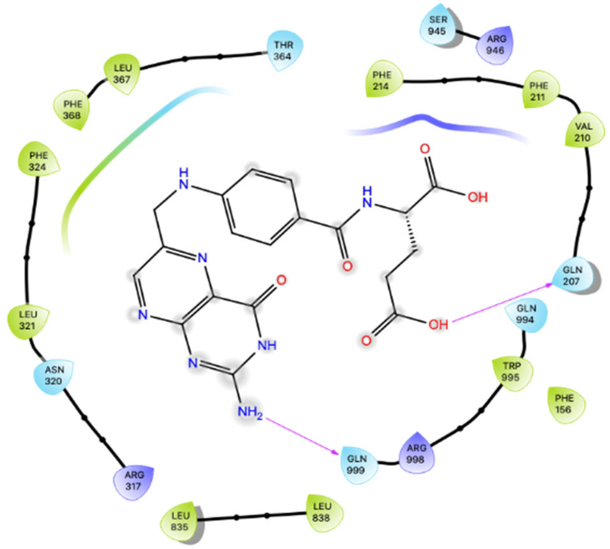


S18. Y556C-FA complex at T0 in AA-MDS

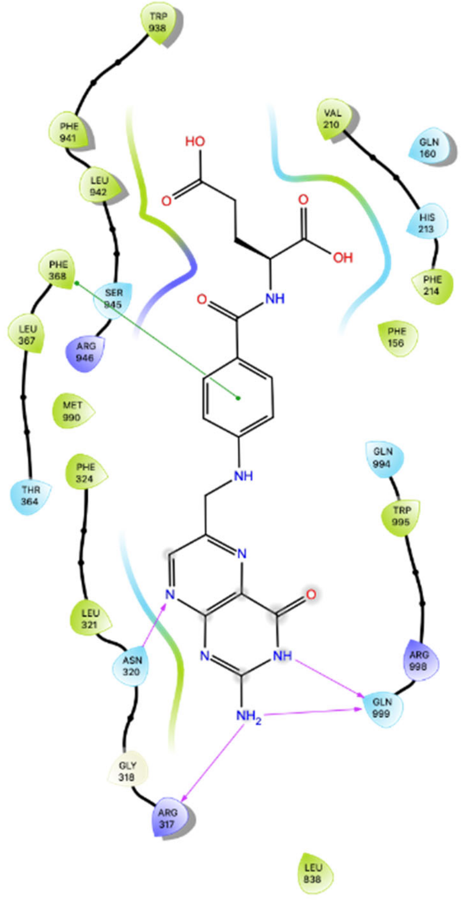


S19. Y556C-FA complex at 5 ns in AA-MDS

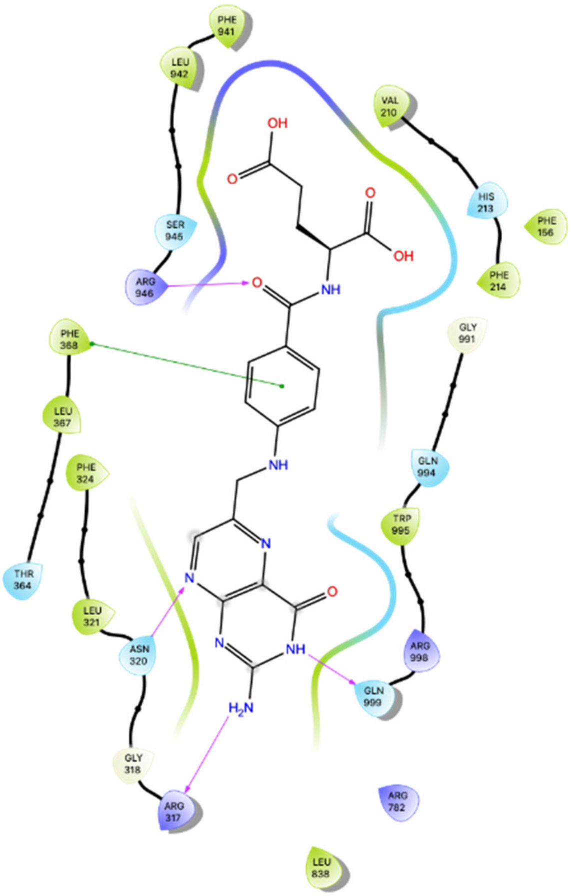


S20. Y556C-FA complex at 10 ns in AA-MDS

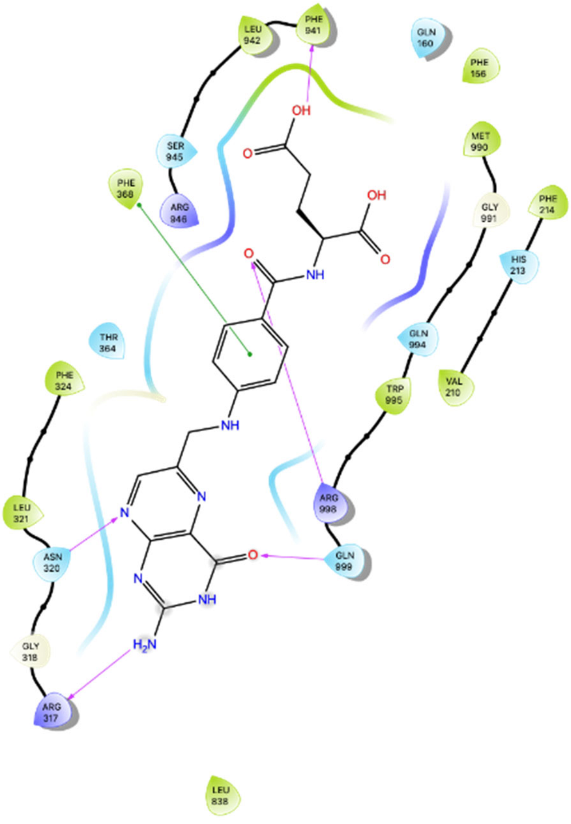


S21. Y556C-FA complex at 15 ns in AA-MDS

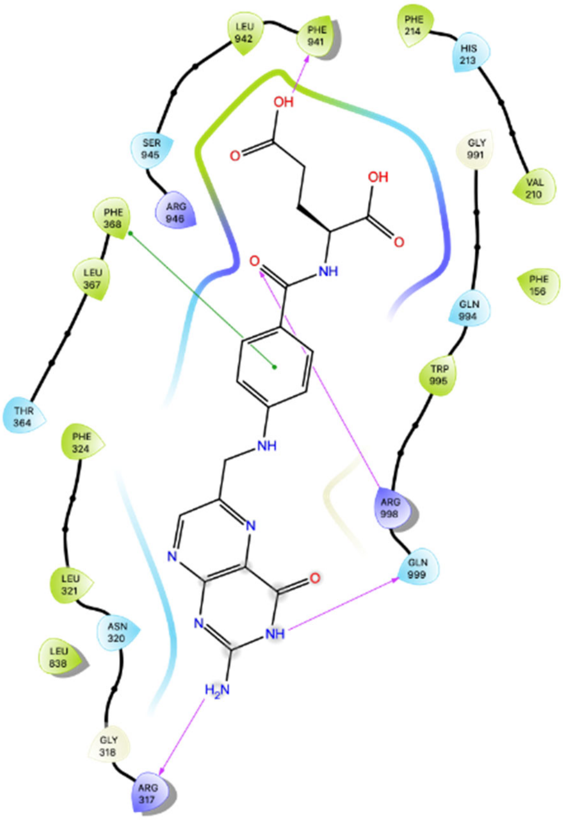


S22. Y556C-FA complex at 20 ns in AA-MDS

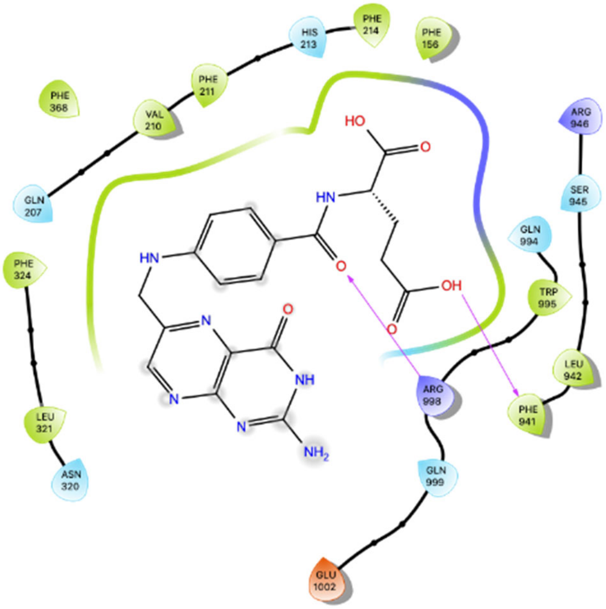


S23. Y556C-FA complex at 25 ns in AA-MDS
